# The RhlR quorum-sensing receptor controls *Pseudomonas aeruginosa* pathogenesis and biofilm development independently of its canonical homoserine lactone autoinducer

**DOI:** 10.1371/journal.ppat.1006504

**Published:** 2017-07-17

**Authors:** Sampriti Mukherjee, Dina Moustafa, Chari D. Smith, Joanna B. Goldberg, Bonnie L. Bassler

**Affiliations:** 1 Princeton University, Department of Molecular Biology, Princeton, NJ, United States of America; 2 Emory University School of Medicine, Children’s Healthcare of Atlanta, Inc., Department of Pediatrics, and Center for Cystic Fibrosis and Airway Diseases Research, Atlanta, GA, United States of America; 3 Howard Hughes Medical Institute, Chevy Chase, MD, United States of America; University of Maryland, UNITED STATES

## Abstract

Quorum sensing (QS) is a bacterial cell-to-cell communication process that relies on the production, release, and response to extracellular signaling molecules called autoinducers. QS controls virulence and biofilm formation in the human pathogen *Pseudomonas aeruginosa*. *P*. *aeruginosa* possesses two canonical LuxI/R-type QS systems, LasI/R and RhlI/R, which produce and detect 3OC12-homoserine lactone and C4-homoserine lactone, respectively. Here, we use biofilm analyses, reporter assays, RNA-seq studies, and animal infection assays to show that RhlR directs both RhlI-dependent and RhlI-independent regulons. In the absence of RhlI, RhlR controls the expression of genes required for biofilm formation as well as genes encoding virulence factors. Consistent with these findings, Δ*rhlR* and Δ*rhlI* mutants have radically different biofilm phenotypes and the Δ*rhlI* mutant displays full virulence in animals whereas the Δ*rhlR* mutant is attenuated. The Δ*rhlI* mutant cell-free culture fluids contain an activity that stimulates RhlR-dependent gene expression. We propose a model in which RhlR responds to an alternative ligand, in addition to its canonical C4-homoserine lactone autoinducer. This alternate ligand promotes a RhlR-dependent transcriptional program in the absence of RhlI.

## Author summary

Quorum sensing (QS) is a cell-to-cell communication process that bacteria use to coordinate group behaviors. QS is essential for virulence and biofilm formation in many bacteria including the human pathogen *Pseudomonas aeruginosa*. *P*. *aeruginosa* has high clinical relevance because it has acquired resistance to commonly used antibiotics, and is a priority pathogen on the CDC ESKAPE pathogen list. The urgent need for new antimicrobials to combat *P*. *aeruginosa* infections makes targeting QS for interference an attractive approach. Here, we investigate *P*. *aeruginosa* under biofilm conditions that mimic authentic *P*. *aeruginosa* lifestyles in environmental and medical contexts rather than in traditional laboratory conditions. This strategy enabled us to find that *P*. *aeruginosa* uses a novel QS signal molecule that controls biofilm formation and virulence. The new signal molecule acts together with the long-known QS receptor RhlR. Using physiologic, genetic, and molecular studies, combined with animal models of infection, we characterize the roles of QS components in biofilm formation and virulence. We find that RhlR and the putative new signal molecule are crucial for both traits. Our work suggests that targeting RhlR with small molecule inhibitors could provide an exciting path forward for the development of novel antimicrobials.

## Introduction

Quorum sensing (QS) is a process of bacterial cell-to-cell communication that relies on the production, detection, and response to extracellular signaling molecules called autoinducers [1]. QS allows groups of bacteria to synchronously alter behavior in response to changes in the population density and species composition of the surrounding bacterial community [2,3]. In Gram-negative bacteria, acylated homoserine lactones (AHLs) are common QS autoinducers (reviewed in [4]). Typically, an AHL synthase, usually a LuxI homolog, produces an autoinducer that is bound by a partner transcriptional activator, usually a LuxR homolog. LuxR-AHL complexes regulate expression of genes that underpin group behaviors [5]. LuxR-type proteins contain two domains: an amino-terminal AHL-binding domain and a carboxy-terminal helix-turn-helix (HTH) DNA-binding domain [6,7]. Most LuxR-type receptors require their cognate AHLs to be bound to function, and in some cases, AHL binding is necessary for LuxR-type proteins to fold and thus resist proteolysis [8–10].

**Fig 1:**
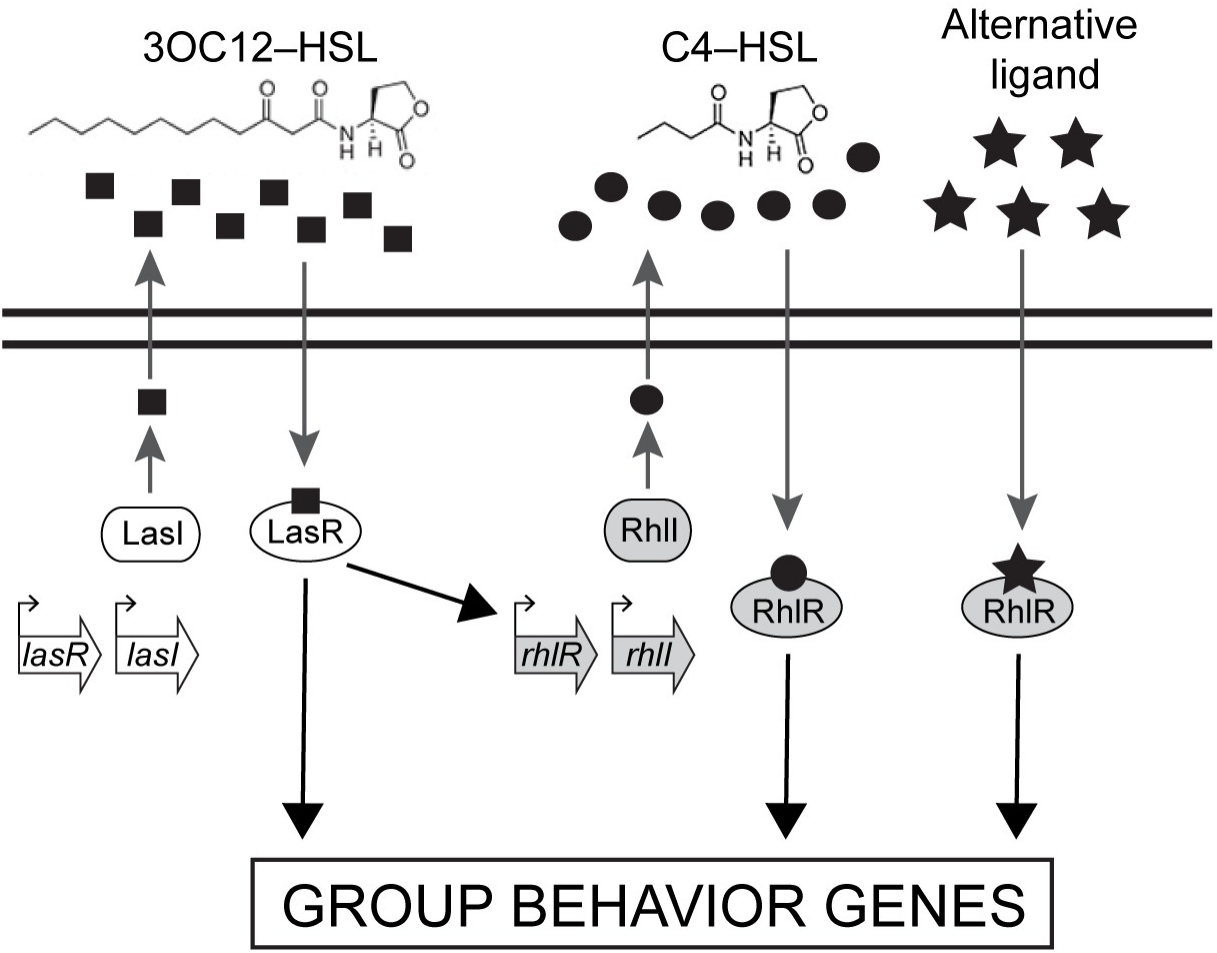
The *Pseudomonas aeruginosa* LuxR/I-type QS circuit. Schematic of the acyl-homoserine lactone (AHL) based QS network: LasR/I (white) and RhlR/I (gray). The LasI autoinducer is 3OC12-HSL (squares) and the RhlI autoinducer is C4-HSL (circles). The two black horizontal lines represent the cytoplasmic membrane, regulatory genes are shown in the open arrows, and bent arrows represent promoters. The alternative ligand (represented by the stars) refers to the putative ligand(s) that binds to RhlR and enables RhlI-independent RhlR function.

Bacterial pathogens often require QS to establish or to promote infection (reviewed in [11]). One such QS bacterium, *Pseudomonas aeruginosa*, is a human pathogen that is frequently responsible for hospital-acquired infections and is the main cause of morbidity and mortality in cystic fibrosis patients [12,13]. The *P*. *aeruginosa* QS circuit consists of two canonical LuxI/R pairs: LasI/R and RhlI/R (Fig 1) [14–17]. LasI produces and LasR responds to the autoinducer *N*-(3-oxododecanoyl)-L-homoserine lactone (3OC12-HSL). The LasR:3OC12–HSL complex activates transcription of many genes including *rhlR* [18]. RhlR binds to the autoinducer *N*-butanoyl-L-homoserine lactone (C4-HSL), the product of the RhlI synthase [19]. RhlR:C4-HSL directs a large regulon of genes including those encoding virulence factors such as pyocyanin, elastases, and rhamnolipids, some of which are also members of the LasR:3OC12-HSL regulon (Fig 1) [20,21]. *P*. *aeruginosa* strains harboring mutations in QS regulatory components have been reported to be attenuated for virulence, and thus, interfering with QS holds promise for the development of novel anti-microbial therapies [22–26].

Beyond controlling virulence, QS controls biofilm formation in *P*. *aeruginosa* [26,27]. Biofilm formation is crucial for *P*. *aeruginosa* acute and chronic infections [29]. QS-activated genes encoding exoproducts such as the Pel and Psl exopolysaccharides, rhamnolipids, and phenazines are key for biofilms because these products drive the architecture of the developing communities [30–33]. In keeping with this overarching role for QS in biofilm formation, in the laboratory, *P*. *aeruginosa lasR* and *lasI* mutants form defective biofilms that are thin, undifferentiated, and easily eradicated by SDS and antimicrobial treatments [27].

Most research examining the role of QS in *P*. *aeruginosa* virulence and biofilm formation has focused on the LasI/R system because of its location at the top of the QS signal transduction cascade (Fig 1). Curiously, however, *lasR* loss of function mutants arise in *P*. *aeruginosa* chronic cystic fibrosis infections [34–36]. Furthermore, QS-controlled virulence traits are expressed in these *lasR* mutants. A possible clue to this conundrum comes from recent work showing that RhlR, not LasR, is the primary QS regulator during host infection, using *Drosophila* as a model [37]. Given this ambiguity, we wanted to define the role of RhlR in virulence and biofilm formation in *P*. *aeruginosa*. In this study, we show that RhlR, previously reported to have an obligate dependence on its canonical AHL autoinducer C4-HSL, can function as a transcriptional regulator in the absence of C4-HSL. Indeed, we show that Δ*rhlR* and Δ*rhlI* mutants have dramatically different biofilm phenotypes. Genome-wide transcriptomics analyses show that RhlR and RhlI likewise control distinct regulons with little overlap under biofilm-forming conditions. We also find that crucial RhlR-regulated virulence factors are expressed in the absence of RhlI. Consistent with this result, the Δ*rhlI* mutant infects nematode and mouse animal hosts as effectively as wildtype *P*. *aeruginosa*, in contrast to the Δ*rhlR* mutant that is attenuated for virulence. Finally, cell-free culture fluids prepared from the Δ*rhlI* mutant possess an activity that stimulates RhlR-dependent gene expression. These findings support the hypothesis that RhlR responds to an alternative ligand, in addition to C4-HSL, and this alternative ligand promotes RhlR-dependent transcriptional activation in the absence of RhlI.

## Results

### *P*. *aeruginosa* Δ*rhlR* and Δ*rhlI* mutants have distinct colony biofilm phenotypes

**Fig 2:**
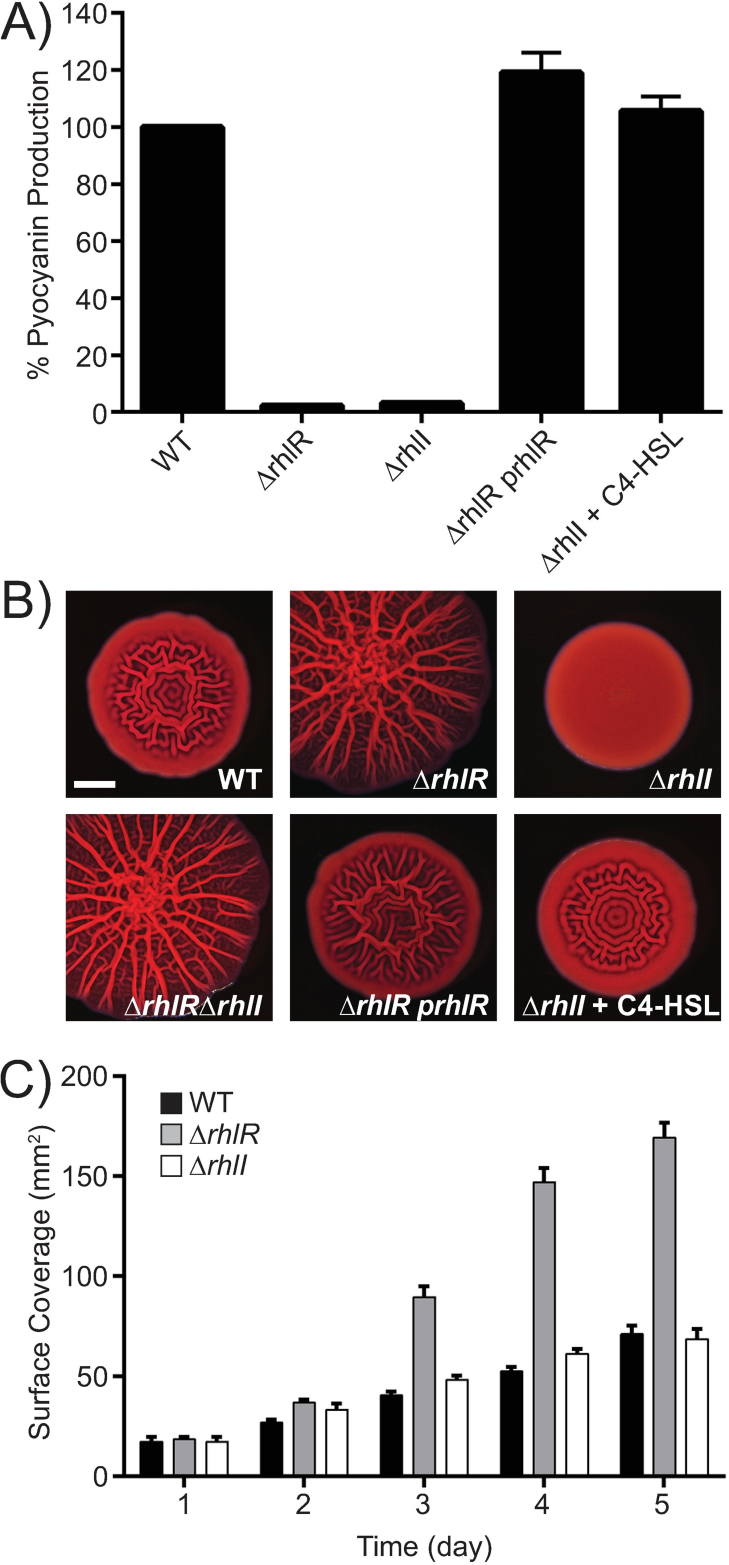
*P*. *aeruginosa* Δ*rhlI* and Δ*rhlR* mutants have distinct colony biofilm phenotypes. A) Pyocyanin production was measured at OD_695_ in cell-free culture fluids prepared from the WT, Δ*rhlR* mutant, Δ*rhlI* mutant, Δ*rhlR* mutant complemented with the *rhlR* gene under its native promoter on pUCP18 (p*rhlR*), and the Δ*rhlI* mutant supplied with exogenous 10 μM C4-HSL. Error bars represent SD for three biological replicates. B) Colony biofilm phenotypes of the strains in panel A and the Δ*rhlR* Δ*rhlI* double mutant. Again, 10 μM C4-HSL was added to the Δ*rhlI* mutant. Scale bar is 2 mm. C) Colony biofilm surface area quantitation for the indicated strains over 5 days. Error bars represent SEM of three independent experiments.

To explore the role of the RhlI/R QS system in *P*. *aeruginosa* virulence and biofilm formation, we generated in-frame marker-less deletions of the *rhlR* and *rhlI* genes in the UCBPP-PA14 strain of *P*. *aeruginosa* (hereafter referred to as PA14). We also made the double Δ*rhlR* Δ*rhlI* mutant. As expected, deletion of *rhlR* or *rhlI* abolished pyocyanin production in planktonic cultures (Fig 2A) [16]. Introduction of a plasmid expressing *rhlR* under its native promoter complemented pyocyanin production in the Δ*rhlR* mutant (Fig 2A). Likewise, exogenous addition of 10 μM C4-HSL to the Δ*rhlI* mutant restored pyocyanin production to wildtype (WT) levels (Fig 2A). Similar results with pyocyanin and other quorum-sensing-controlled phenotypes have been reported previously, including with mutant strains used in the present work [16,38,39,40,41]. These analyses confirm that every component of our system is functional. We note, and we will return to this point later, that similar to other AHL-dependent LuxR-type transcriptional activators, the Δ*rhlR* and Δ*rhlI* mutants phenocopy each other with respect to pyocyanin production in planktonic culture.

*P*. *aeruginosa* PA14 forms biofilms in submerged systems (flow-cell biofilms), at liquid-air interfaces (pellicles), and on solid-air interfaces (colony biofilms) [30,42]. Colony biofilm formation can be studied using agar plates containing Congo red, a dye that binds to extracellular matrix components [30]. On colony biofilm medium, WT *P*. *aeruginosa* PA14 exhibited the characteristic, and previously reported, rugose-center/smooth-periphery colony biofilm phenotype after 5 days of growth (Fig 2B) [31]. To our knowledge, the roles of RhlR and RhlI have not previously been investigated in *P*. *aeruginosa* PA14 colony biofilms. We found that the Δ*rhlR* mutant was hyper-rugose (Fig 2B and S1A Fig) and introduction of *rhlR* on a plasmid restored the WT morphology (Fig 2B). We conclude that RhlR controls colony biofilm development. Surprisingly, the Δ*rhlI* mutant had a phenotype that was strikingly different from the WT and the Δ*rhlR* mutant. The Δ*rhlI* mutant was completely smooth, indeed, even more so than the WT (Fig 2B; S1A Fig). Exogenous addition of 10 μM C4-HSL to the agar medium restored the WT biofilm phenotype to the Δ*rhlI* mutant (Fig 2B). The Δ*rhlR* phenotype is epistatic to the Δ*rhlI* phenotype because the Δ*rhlR* Δ*rhlI* double mutant phenocopies the Δ*rhlR* single mutant (Fig 2B; S1B Fig). This phenotypic difference also occurred on agar plates lacking any dyes, confirming that the distinct morphologies of the Δ*rhlR* and Δ*rhlI* mutants are not a consequence of the Congo red medium (S1C Fig). The Δ*rhlR* mutant colony biofilms expanded to cover more surface area than did those of the WT and the Δ*rhlI* mutant (Fig 2C; S1A Fig). These results are in stark contrast to those for the LasI/R system: we constructed marker-less in-frame Δ*lasR* and Δ*lasI* mutants and found that both mutants have identical pyocyanin, colony biofilm, and surface coverage phenotypes (S2A, S2B and S2C Fig).

A previous genome-wide small RNA-seq study identified a putative trans-acting sRNA called SPA0104 in *P*. *aeruginosa* PA14 [43]. The *SPA0104* gene is located between the *rhlR* and *rhlI* genes, co-oriented and overlapping with the *rhlI* promoter [43]. To determine if SPA0104 is involved in the different Δ*rhlR* and Δ*rhlI* phenotypes we observed above, we engineered stop codons into the *rhlR* and *rhlI* genes: *rhlR*^*W11STOP*^ and *rhlI*^*F50STOP*^. Our rationale was that insertion of a stop codon would prevent translation of the full-length RhlR or full length RhlI protein without affecting transcription of *SPA0104*, enabling us to determine if the SPA0104 sRNA contributed to the biofilm phenotypes. Neither of the mutants produced pyocyanin showing that, in the case of RhlR and RhlI, introduction of the stop codon eliminated function (S2A Fig). Nonetheless, the *rhlR*^*W11STOP*^ mutant was hyper-rugose and the *rhlI*^*F50STOP*^ mutant was completely smooth in the colony biofilm assay (S2D Fig). We therefore conclude that the Δ*rhlR* and Δ*rhlI* mutants have distinct colony biofilm phenotypes and the SPA0104 sRNA plays no role in conferring these phenotypes.

Rhamnolipids, RhlR-activated exoproducts, have been reported to disperse biofilms [44,45]. Thus, it was possible that, in the Δ*rhlR* mutant, the absence of rhamnolipids decreased dispersal, and this defect in the process conferred the hyper-rugose phenotype. If so, disabling rhamnolipid production should cause a hyper-rugose phenotype irrespective of the presence or absence of RhlR. We investigated this possibility by inactivating the rhamnolipid biosynthetic gene *rhlA* [46,47] via introduction of a stop codon (*rhlA*^*C11STOP*^). The *rhlA*^*C11STOP*^ mutant has a colony biofilm phenotype that is indistinguishable from the WT, so it is not hyper-rugose (S3 Fig). By contrast, the Δ*rhlR rhlA*^*C11STOP*^ double mutant is hyper-rugose, and the Δ*rhlI rhlA*^*C11STOP*^ double mutant is completely smooth (S3 Fig). We therefore conclude that rhamnolipids are not involved in the distinct colony biofilm phenotypes we have discovered for the Δ*rhlR* and Δ*rhlI* mutants.

### RhlR and RhlI control distinct but overlapping regulons

**Fig 3:**
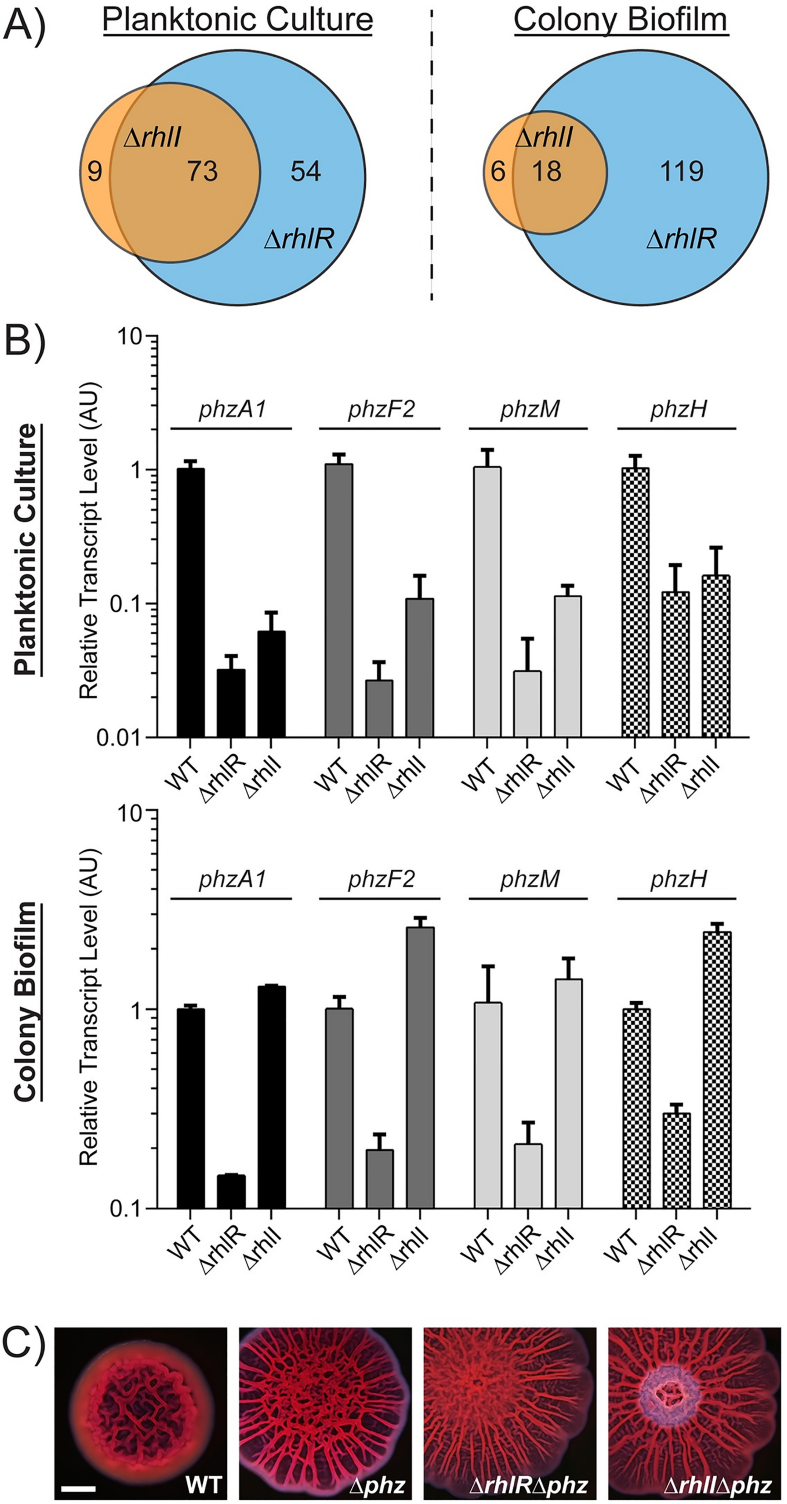
*P*. *aeruginosa* has distinct RhlR and RhlI regulons. A) Venn diagram showing differentially expressed genes in the Δ*rhlI* and Δ*rhlR P*. *aeruginosa* PA14 strains at high cell density (denoted Planktonic Culture; OD_600_ = 2.0) and in colony biofilms grown on Congo red agar medium for 5 days (denoted Colony Biofilm). Blue circles represent differentially expressed genes in the Δ*rhlR P*. *aeruginosa* strain compared to the WT strain. Orange circles indicate differentially expressed genes in the Δ*rhlI P*. *aeruginosa* strain compared to the WT strain. Numbers of cDNA reads for annotated genes were compared to the WT strain under the same conditions. Statistically significant genes (P < 0.001) that changed >2-fold are shown (see S1 and S2 Tables). RNA-seq was performed on three biological replicates per strain and per condition. B) Relative expression of the representative phenazine biosynthesis genes *phzA1* (black), *phzF2* (dark gray), *phzM* (light gray), and *phzH* (checkered). Data are normalized to 16S RNA measured by qRT-PCR in WT, Δ*rhlR*, and Δ*rhlI* strains in high cell density (OD_600_ = 2.0) planktonic culture (top panel) and in colony biofilms on Congo red agar medium after 5 days (bottom panel). Error bars represent SD of three replicates. AU denotes arbitrary units. C) Colony biofilm morphology of the WT, Δ*phz*, and the Δ*rhlR* Δ*phz*, and Δ*rhlI* Δ*phz* mutants. Scale bar is 2 mm.

To define the molecular basis underpinning the different Δ*rhlR* and Δ*rhlI* colony biofilm phenotypes, we used RNA-seq to compare the genomic transcriptional profiles of the WT, Δ*rhlR*, and Δ*rhlI* strains. We performed the experiment under two conditions: on mRNA harvested from high cell density (HCD) planktonic cultures and on mature colony biofilms. To our knowledge, this is the first transcriptional profiling study performed on the Δ*rhlI* mutant. The results from HCD planktonic cultures match previously published studies for the WT and the Δ*rhlR* mutant [18,26]. Roughly 127 genes constitute the RhlR regulon, defined as greater than two-fold changes in expression in the Δ*rhlR* mutant compared to the WT in HCD planktonic cultures (Fig 3A, S1 Table). Seventy-three of those genes were also regulated by RhlI. Under colony biofilm forming conditions, the Δ*rhlR* mutant exhibited differences in expression of 137 genes compared to the WT. However, only 18 of those genes showed more than two-fold changes in the Δ*rhlI* mutant relative to the WT (Fig 3A; S2 Table). Thus, of the RhlR-regulated genes, ~54% were in common between the Δ*rhlR* and Δ*rhlI* mutants in HCD planktonic cultures. By contrast, there was only ~13% overlap in the RhlR-directed gene expression profiles in these two mutants in colony biofilms (Fig 3A; S1 and S2 Tables). We note that 9 and 6 genes were uniquely regulated by RhlI under planktonic and colony biofilm formation conditions, respectively. A few autoinducer-dependent, receptor-independent QS-regulated genes have been reported previously for the *P*. *aeruginosa* Las system [40,48]. In summary, RhlR regulates the expression of numerous genes independently of its canonical AHL autoinducer C4-HSL.

The most striking difference between the transcriptional profiles of the Δ*rhlR* and Δ*rhlI* mutants in planktonic and colony biofilm culture conditions concerned the phenazine (*phz*) biosynthesis genes (S1 and S2 Tables). Specifically, each *phz* gene exhibited 10-fold lower expression in both the Δ*rhlR* and Δ*rhlI* mutants compared to the WT in HCD planktonic culture (Fig 3B). In colony biofilms, the Δ*rhlR* mutant continued to exhibit 10-fold lower expression of *phz* genes compared to WT. However, the Δ*rhlI* mutant expressed the *phz* genes at WT levels (Fig 3B).

Endogenously produced phenazines act as redox-active small molecules to modulate *P*. *aeruginosa* colony biofilm morphogenesis. Specifically, mutants that overproduce phenazines have smooth morphologies, whereas mutants that are unable to produce phenazines are hyper-rugose compared to the WT [30,31,33]. We suggest that the Δ*rhlI* mutant has a smooth colony biofilm phenotype due to overproduction of phenazines while the Δ*rhlR* mutant forms a hyper-rugose biofilm colony due to the lack of phenazines. We verified this idea using two different approaches. First, we deleted the two phenazine biosynthesis operons (Δ*phz1* and Δ*phz2*; we call this double mutant Δ*phz*) in the WT and in the Δ*rhlR* and Δ*rhlI* mutant backgrounds. All of the Δ*phz* mutants were hyper-rugose under colony biofilm growth conditions (Fig 3C). Second, we quantified pyocyanin production from the WT, the Δ*rhlR* and Δ*rhlI* single mutants, and the Δ*rhlI* Δ*phz* double mutant when grown as colony biofilms. The wild-type colony biofilm produced ~ 2 μg /10^5^ CFU pyocyanin, the Δ*rhlI* colony biofilm produced four-fold more pyocyanin, while the Δ*rhlR* mutant and the Δ*rhlI* Δ*phz* double mutant produced none (S4 Fig). Together, the deletion analysis and pyocyanin production results show that the Δ*rhlI* smooth colony biofilm phenotype is caused by overproduction of phenazines.

In *P*. *aeruginosa* PA14, the hyper-rugosity conferred by the absence of phenazines requires Pel, the primary biofilm matrix exopolysaccharide (note: *P*. *aeruginosa* PA14 does not produce the Psl exopolysaccharide) [28,30,32]. We examined the colony biofilm phenotypes of the Δ*pelA* single and the Δ*rhlR*Δ*pelA* and Δ*rhlI*Δ*pelA* double mutants. All of these mutants had completely smooth colony morphologies (S5 Fig). Thus, the Pel exopolysaccharide is required for the Δ*rhlR* mutant to exhibit the hyper-rugose biofilm phenotype. We note that the mechanism by which the overproduction of phenazines downregulates Pel to cause the smooth colony biofilm phenotype is unknown and beyond the scope of this study. What is crucial for our work is that the Δ*rhlI* mutant retains the ability to produce phenazines in colony biofilms while the Δ*rhlR* mutant does not.

### RhlR regulon genes exhibit discrete levels of RhlI dependence

**Fig 4:**
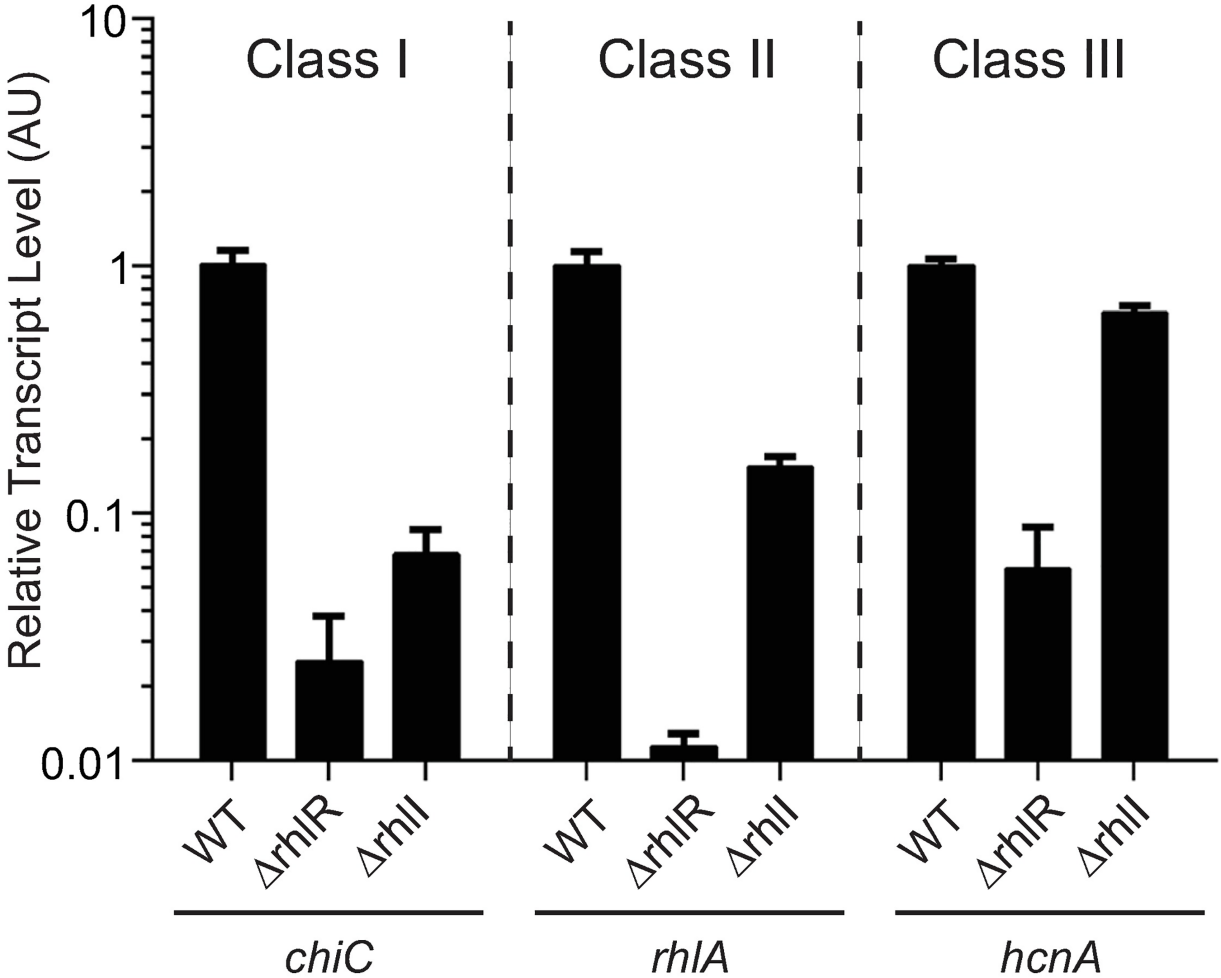
RhlR-activated genes exhibit varying levels of RhlI dependence. Relative expression of the *chiC*, *rhlA*, and *hcnA* genes normalized to 16S RNA measured by qRT-PCR in the WT, Δ*rhlR*, and Δ*rhlI* strains at high cell density (OD_600_ = 2.0). Class I, II, III denote RhlI-dependent, partially dependent, and independent genes, respectively. AU denotes arbitrary units. Error bars represent SD of three replicates.

Close inspection of the RNA-seq data from the HCD planktonic cultures (S1 Table) revealed three classes of RhlR-regulated genes based on their dependence on RhlI: genes which we call Class I, exemplified by *chiC*, that require RhlI for RhlR-mediated activation; Class II genes, represented by *rhlA*, that require RhlR for activation but are only partially RhlI dependent, and Class III genes, such as *hcnA*, that require RhlR for activation but are RhlI independent. Fig 4 shows quantitative RT-PCR results for the representative genes and complementation assays are shown in Fig 2 and S6 Fig. Based on this classification scheme, the phenazine biosynthesis genes exhibit Class I behavior in HCD planktonic cultures but they behave as Class III genes in colony biofilms. We infer that an as yet unknown ligand(s) exists that allows RhlR to function independently of RhlI in colony biofilms (Fig 1). We speculate that this ligand increases during biofilm formation and promotes RhlR-dependent activation of *phz* transcription in the absence of the canonical, RhlI-produced autoinducer C4-HSL (Fig 3B).

### Cell-free culture fluids from the Δ*rhlI* mutant activate RhlR-dependent gene expression

**Fig 5:**
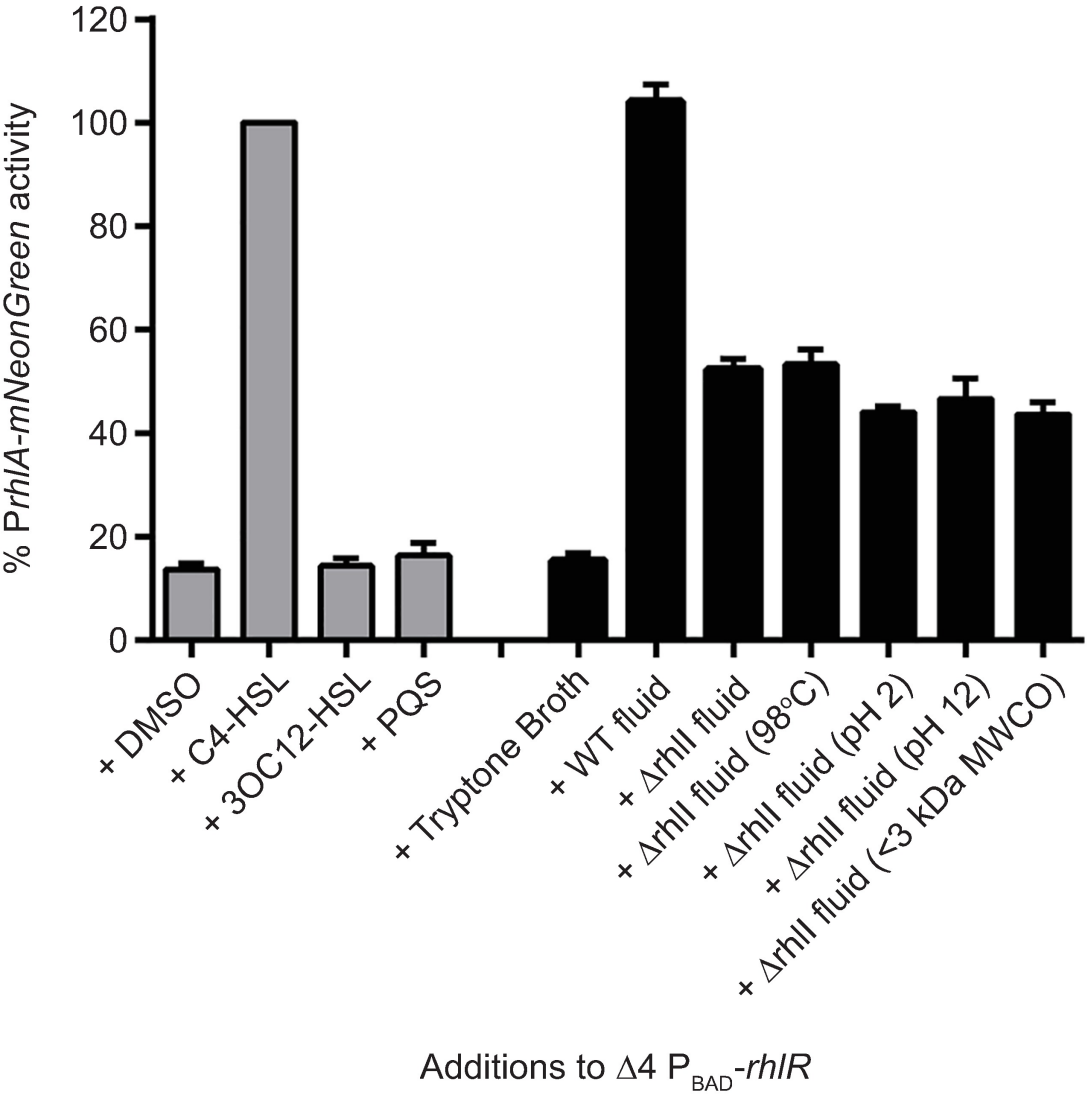
Cell-free culture fluids from the Δ*rhlI* mutant activate RhlR-dependent gene expression. *rhlA* expression was measured using a chromosomally encoded P*rhlA- mNeonGreen* transcriptional reporter. Gray bars represent *rhlA* reporter activity when *rhlR* was induced in the Δ*lasR* Δ*lasI* Δ*rhlR* Δ*rhlI* (i.e., Δ4 P_BAD_-*rhlR*) strain with 0.1% L-arabinose in the presence of 1% DMSO, 10 μM C4-HSL, 10 μM 3OC12-HSL, or 50 μM PQS. DMSO was used as the solvent for C4-HSL, 3OC12-HSL, and PQS. The *rhlA* reporter activity was set to 100% when 10 μM C4-HSL was added. In the cultures represented by the black bars, P*rhlA-mNeonGreen* was monitored in response to 20% (v/v) of the indicated cell-free culture fluids subjected to the specified treatments. Error bars represent SEM for three biological replicates.

To garner evidence for a new ligand that acts in conjunction with RhlR, we made a fluorescent transcriptional reporter fusion to the *rhlA* promoter (P*rhlA-mNeonGreen*). We chose to follow *rhlA* because it is a Class II gene (Fig 4), and thus, is expressed in a RhlR-dependent manner both in the presence and absence of RhlI. We incorporated the reporter fusion into an intergenic region on the chromosomes of WT *P*. *aeruginosa* and the Δ*rhlR* and Δ*rhlI* mutants. The P*rhlA-mNeonGreen* reporter exhibited 10-fold lower expression in the Δ*rhlR* mutant than the WT (set to 100%) in HCD planktonic culture (S7A Fig). Expression of the reporter in the Δ*rhlI* mutant was reproducibly 30% of that in the WT (S7A Fig). These results show that, first, RhlR is absolutely required for expression of P*rhlA-mNeonGreen* and, second, the reporter fusion continues to be expressed when RhlR is present but RhlI (i.e., C4-HSL) is not. By contrast, the Δ*lasR* and Δ*lasI* mutants both exhibit low, but identical, reporter activity (S7A Fig). Thus, LasR does not control *rhlA* in the absence of LasI (i.e., 3OC12-HSL). Next, we built a strain in which we deleted the *lasR*, *lasI*, *rhlR*, and *rhlI* genes (called Δ4) and inserted an arabinose-inducible *rhlR* gene at the *glmS* locus. We call this strain Δ4 P_BAD_-*rhlR*. We inserted the P*rhlA-mNeonGreen* transcriptional reporter fusion onto the chromosomes of the Δ4 and the Δ4 P_BAD_-*rhlR* strains. Both the Δ4 mutant and the Δ4 P_BAD_-*rhlR* strain showed background level reporter activity similar to the Δ*rhlR* mutant (S7A Fig). Exogenous addition of DMSO to the Δ4 P_BAD_-*rhlR* strain in conjunction with L-arabinose induction of *rhlR* also resulted in background activity, which indicates that unliganded RhlR is not capable of activating gene expression (Fig 5, gray bars). Addition of 10 μM synthetic C4-HSL, along with L-arabinose induction of *rhlR*, restored the WT level of reporter activity. We set this level of activity to 100%. Addition of 10 μM synthetic 3OC12-HSL or 50 μM synthetic PQS (2-heptyl-3-hydroxy-4-quinolone, a third *P*. *aeruginosa* QS autoinducer that functions together with a receptor called PqsR [49]) to the Δ4 P_BAD_-*rhlR* strain, along with L-arabinose, did not activate reporter activity above background levels (Fig 5, gray bars). Thus, neither 3OC12-HSL nor PQS activate RhlR-driven transcription of *rhlA*, and so those QS molecules cannot be responsible for the activity present in the cell-free culture fluids prepared from the Δ*rhlI* mutant.

We next determined whether the activity driving RhlR-dependent expression of the P*rhlA-mNeonGreen* reporter fusion in the Δ*rhlI* mutant is present in cell-free culture fluids. Exogenous addition of 20% (v/v) WT cell-free culture fluids to the Δ4 P_BAD_-*rhlR* strain in conjunction with L-arabinose induction of *rhlR* resulted in 10-fold higher reporter activity compared to the background activity obtained following the addition of medium (Fig 5, black bars). Addition of cell-free culture fluids from the Δ*rhlI* mutant stimulated 55% of the activity stimulated by WT cell-free culture fluids, consistent with the idea that a ligand is indeed present in Δ*rhlI* culture fluids that can activate RhlR (Fig 5). We considered the possibility that the Δ*rhlI* mutant produced C4-HSL by a non-RhlI-mediated mechanism. However, LC-MS analyses showed that C4-HSL was present in WT cell-free cultures fluids whereas none could be detected in fluids from the Δ*rhlI* mutant (S8 Fig), eliminating this formal possibility. We undertook an initial characterization of the activity. It is stable to high temperature, acidic and basic conditions, and it passes through a 3 kDa size exclusion filter (Fig 5). These results preliminarily suggest that the alternative ligand is a small molecule that is not a homoserine lactone (homoserine lactones are not base or heat stable, [50]). We also tested whether phenazines could be the alternate ligand. To do this, we supplied cell-free culture fluids from the Δ*rhlI* Δ*phz* mutant to the *PrhlA-mNeonGreen* reporter strain and compared the fluorescence output to that when cell-free culture fluids from the Δ*rhlI* mutant were supplied. Both preparations elicited the same amount of reporter activity (S7B Fig) showing that phenazines are not capable of activating RhlR-driven gene expression. We conclude that the Δ*rhlI* mutant cell-free culture fluids contain an as-yet-unidentified ligand(s) that is capable of activating RhlR-dependent target gene expression.

### RhlR functions independently of RhlI in animal infection models

**Fig 6:**
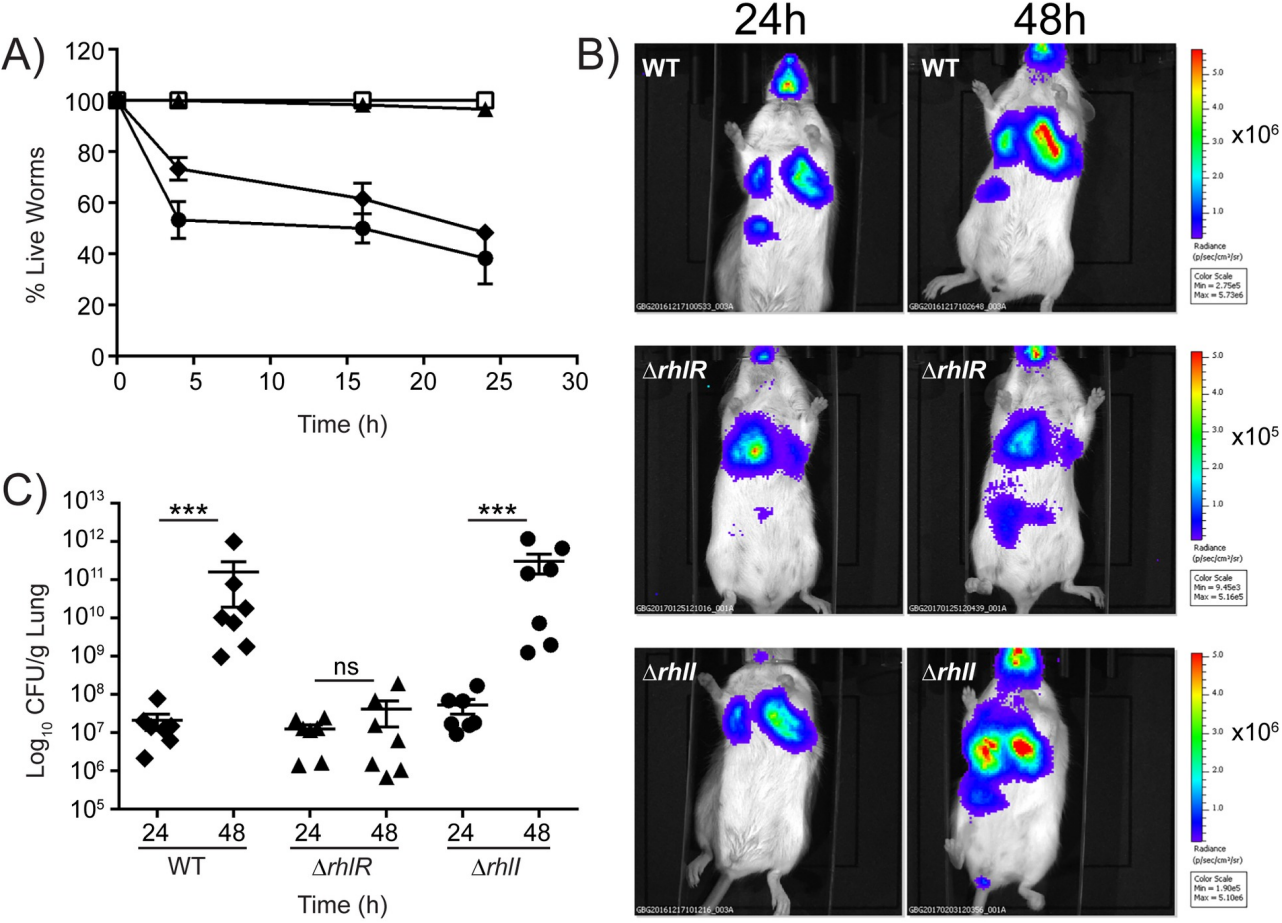
The *P*. *aeruginosa* Δ*rhlI* mutant is virulent and the Δ*rhlR* mutant is avirulent in animal infection models. A) *C*. *elegans* were applied to lawns of *E*. *coli* OP50 (open squares), WT *P*. *aeruginosa* PA14 (closed diamonds), Δ*rhlR* mutant (closed triangles), and Δ*rhlI* mutant (closed circles). Error bars represent SEM of three independent replicates. B) Real-time monitoring of WT *P*. *aeruginosa* PA14 *P1-lux* and isogenic mutants in the acute pneumonia model. BALB/c mice infected with WT, Δ*rhlR*, and Δ*rhlI* strains were imaged at 24 and 48 h using an IVIS CCD camera following intratracheal infection. Imaging was performed from the ventral side of representative mice while the animals were under isoflourane anesthesia. The color bars indicate the intensity of the bioluminescence output, with red and blue denoting the high and low signals, respectively. Note that the color scales on the various mouse bioluminescence imaging panels are not the same. (C) Bacterial load in lung homogenates of mice infected intratracheally with WT *P*. *aeruginosa* PA14, and the Δ*rhlR* and Δ*rhlI* mutants. Each symbol represents a single mouse. The data are pooled from two independent experiments. Data were analyzed using the Mann-Whitney U test. *** P <0.001 and ns means not significant.

Our finding that RhlR is active in the absence of RhlI suggested to us that there could be RhlI-independent RhlR function under non-laboratory conditions such as during host infection. To probe this possibility, we assessed the relative pathogenicity of WT, Δ*rhlR*, and Δ*rhlI P*. *aeruginosa* PA14 strains in a *Caenorhabditis elegans* fast-kill infection assay [26,51]. The Δ*rhlR* mutant was completely avirulent in this assay, while the Δ*rhlI* mutant remained fully virulent, killing worms as proficiently as WT *P*. *aeruginosa* PA14 (Fig 6A). By contrast, both the Δ*lasR*, and Δ*lasI* mutants were as virulent as the WT (S9A Fig). These results demonstrate that RhlR does indeed function in the absence of the canonical C4-HSL autoinducer to control virulence in nematodes. Phenazines mediate *C*. *elegans* killing in the fast-kill infection assay. To determine if the overproduction of phenazines in the Δ*rhlI* strain was responsible for the virulence phenotype, we deleted the two phenazine biosynthetic gene clusters, *phzA1-G1* and *phzA2-G2*, in the Δ*rhlI* mutant (called Δ*rhlI* Δ*phz*). The Δ*rhlI* Δ*phz* mutant was attenuated for virulence similar to the Δ*phz* mutant (S10 Fig) showing that the production of phenazines underpins pathogenicity in the Δ*rhlI* strain.

We also examined the roles of the different QS regulators in a Balb/c murine model of acute *P*. *aeruginosa* lung infection. The 50% lethal doses (LD_50_) for the WT, Δ*rhlR*, Δ*rhlI*, Δ*lasR*, and Δ*lasI P*. *aeruginosa* PA14 strains were 1.90 x 10^6^ CFU, 2.6 x 10^6^ CFU, 1.1 x 10^6^ CFU, 3.0 x 10^6^ CFU, and 4.8x10^6^ CFU, respectively. To assess the effects of these mutations on virulence in the murine model, we monitored the time-dependence of infection following intratracheal challenge with sublethal doses (~0.5 LD_50_) of the strains under study. All of the strains carried a constitutively-expressed *luxCDABE* operon that had been inserted in the chromosome at the *glmS* locus, which enabled real-time monitoring of bacterial colonization of the mouse during the course of infection using an IVIS Imaging System. At 24 h, comparable levels of bioluminescence were detected, primarily in the lungs, in all of the infected mice. At 48 h, however, the signal was approximately 20-fold stronger in mice infected with the WT and the Δ*rhlI* strain than in mice infected with the Δ*rhlR*, Δ*lasI*, and Δ*lasR* strains (Fig 6B; S9B Fig). The imaging data were validated by determining the viable *P*. *aeruginosa* CFU per gram of lung homogenate. At 24 h post-infection, all of the infected mice had similar bacterial burdens. The average CFU/gram were 2.1 X 10^7^ for WT, 1.2 X 10^7^ for the Δ*rhlR* strain, and 5.2 X 10^7^ for the Δ*rhlI* strain (Fig 6C). At 48 h post-infection, the bacterial load in the lungs of mice infected with the WT and Δ*rhlI* mutant had increased to 1.6 X 10^11^ and 3.1 X 10^11^ CFU/gram, respectively (Fig 6C). By contrast, the bacterial load in lungs of mice infected with the Δ*rhlR* strain did not increase significantly between 24 and 48 h, maintaining an average of 4.1 X 10^7^ CFU/ gram. Thus, the WT and Δ*rhlI* mutant produce a four-order of magnitude larger burden of bacteria in the murine host than does the Δ*rhlR* strain. Unlike in the *C*. *elegans* infection assay, in the murine model of acute lung infection, the Δ*lasI* and Δ*lasR* mutants did not display the same level of virulence as the WT, but, rather, were attenuated. Specifically, at 48 h, mice infected with the Δ*lasI* and Δ*lasR* strains maintained the same bacterial load as at 24 h (S9C Fig). We discuss these differences below. In summary, our results show that RhlR is active in the absence of the RhlI-produced autoinducer in both the *C*. *elegans* and the murine animal models. Presumably, a ligand, other than C4-HSL, promotes RhlR function in these two animal assays.

## Discussion

*P*. *aeruginosa* uses its two major LuxI/R QS systems, the Las and Rhl systems, to orchestrate the synchronous production of virulence factors and to form biofilms [52]. Both LasR and RhlR regulate gene expression at high cell density when bound to their cognate AHLs. Both receptors recognize DNA motifs possessing dyad symmetry called *las-rhl* boxes, and both receptors are global transcriptional regulators [20,21]. Here we show, for the first time, that unlike LasR that requires its canonical 3OC12-HSL autoinducer to regulate transcription, RhlR, controls gene expression in both a C4-HSL-dependent and C4-HSL-independent manner. Importantly, C4-HSL-independent regulation by RhlR appears to be highly relevant in biofilms and is critical for pathogenicity in animal models of *P*. *aeruginosa* infection.

One mechanism by which RhlR could function in the absence of RhlI is if RhlR activates transcription of target genes without bound ligand. Indeed, LuxR-type regulators with such capability exist. Unliganded EsaR from *Pantoea stewartii*, ExpR from *Erwinia chrysanthemi*, and VirR from *E*. *carotovora* adopt active dimeric conformations and repress transcription [53–55]. However, RhlR is insoluble, and thus must be inactive, when not bound to a ligand [26]. Consistent with this notion, RhlR displays no basal activity in the absence of exogenously supplied C4-HSL in an *E*. *coli* overexpression assay [41]. While it remains a formal possibility that RhlR functions in the absence of a ligand, another possibility that is more consistent with our data and with what is known about RhlR is that an alternative ligand exists that enables RhlR to act as a transcription factor. Our finding that cell-free culture fluids from the Δ*rhlI* mutant activate RhlR-dependent gene expression strongly supports the alternative ligand hypothesis. We are currently working to purify and identify this putative ligand. The alternative ligand does not appear to be an AHL: the *P*. *aeruginosa* genome contains only two LuxI homologs, LasI and RhlI, and the LasI product 3OC12-HSL does not stimulate RhlR-dependent gene expression (Fig 5). Furthermore, 3OC12-HSL is known to inhibit RhlR activity [38]. The alternative ligand is stable in alkaline pH and at high temperatures while canonical AHLs are not [49]. A non-AHL ligand made by a non-LuxI-type synthase is also a possibility, and consistent with this notion, the LuxR homologs PluR and PauR in *Photorhabdus* spp. bind to pyrones and dialkylresorcinols, respectively, to regulate target gene expression [56,57]. As far as is known, PluR and PauR do not bind AHLs.

LuxR-type proteins exhibit a spectrum of specificities for ligands. Some LuxR-type proteins, including LasR and TraR, are exquisitely specific for their cognate autoinducers [6,58]. Others, including SdiA and CviR, are promiscuous and bind to a variety of AHLs [59,60]. Ligand recognition promiscuity could be a mechanism that enhances the versatility of QS systems in different niches. By expanding the repertoire of stimuli detected, and linking the binding of particular ligands by a LuxR-type receptor to expression of particular subsets of target genes, bacteria could reuse their LuxR-type receptors to tune into and successfully colonize different habitats. Such a scenario would endow *P*. *aeruginosa* with the plasticity to diversify its QS outputs, while also being especially economical because it does not necessitate the evolution of a new transcription factor for every small molecule stimulus that is detected. Indeed, *P*. *aeruginosa* occupies diverse environments including soil, marshes, plants and animals, and QS is required for success in all of these niches [61,62].

It is possible that additional ligands exist for RhlR, and perhaps for LasR, beyond the new activity we have uncovered here for RhlR. We suggest that each such a ligand or sets of ligands are vital in particular niches making their discovery difficult under laboratory conditions. Indeed, our experiments revealed the activity of one putative alternative RhlR ligand only because we examined colony biofilm conditions, rather than traditional liquid growth. Consistent with this assertion, RNA-seq profiles from *P*. *aeruginosa* HCD planktonic cultures and colony biofilms show that RhlR is dramatically less dependent on RhlI, and thus the C4-HSL ligand, in colony biofilms than in planktonic culture. We speculate that the concentration of our proposed alternative ligand increases in colony biofilms enabling the transition of RhlR from being predominantly bound to C4-HSL to being predominantly bound to the alternative ligand. This change, in turn, promotes the transition from expression of RhlR-directed Class I to RhlR-directed Class II and Class III target genes. In this respect, RhlR resembles the multiple antibiotic resistance regulator MarR and homologs from diverse bacteria and nuclear receptors from higher eukaryotes that bind multiple ligands, and depending on which ligand is bound, control expression of discrete subsets of genes [63,64].

Phenazine production contributes to virulence in diverse *P*. *aeruginosa* infection models [33,52,65]. Consistent with this finding, we showed that the Δ*rhlI* mutant that produces phenazines is virulent in both a nematode and a murine infection model while the Δ*rhIR* mutant, which is defective for phenazine production, is attenuated (Fig 6). Our finding that the Δ*lasR and* Δ*lasI* mutants produce phenazines in nematode assay medium provides one condition in which the Rhl system bypasses the requirement for the Las system to promote downstream QS target gene expression (S11 Fig). Phenazine production presumably enables the Δ*lasR and* Δ*lasI* mutants to exhibit WT level virulence in the *C*. *elegans* fast-kill infection assay. We do note, however, that the Δ*lasR and* Δ*lasI* mutants fail to establish a WT level of infection in the murine acute pneumonia model suggesting that a factor(s) other than phenazines plays a crucial role in virulence in mice.

Previous studies have examined QS control of *P*. *aeruginosa* virulence in a mouse burn model [13], a corneal infection model [66], a murine neonatal model of acute pneumonia [67], and mouse and rat models of chronic lung infection [68,69]. The majority of these earlier studies were performed using *las* mutants or strains containing both *las* and *rhl* mutations. Rumbaugh *et al* [13] reported that the *P*. *aeruginosa* PA14 Δ*rhlI* mutant was significantly less virulent than the WT in a mouse burn model. Our results show that the *P*. *aeruginosa* PA14 Δ*rhlI* single mutant is as virulent as WT in a murine acute pneumonia model. Tissue specific responses to different *P*. *aeruginosa* PA14 virulence factors could explain these different results.

Our *in vivo* imaging of *P*. *aeruginosa* following intratracheal infection of BALB/c mice revealed infection beyond the respiratory tract (Fig 6B; S9B Fig). The components driving dissemination are not currently known. Earlier rodent studies examining respiratory infection by *P*. *aeruginosa* PA14 did not monitor infection in other organs [70,71]. *P*. *aeruginosa* PA14 produces the cytotoxin ExoU [72], potentially enabling endothelial permeability and systemic escape of bacteria from the mouse lung into the periphery. However, whether ExoU is responsible for the dissemination from the mouse lung has not been examined. Going forward, the experimental system we developed in this work provides the opportunity to test ExoU as well as other components for roles in *P*. *aeruginosa* PA14 dissemination and systemic infection burden in a mammalian context.

*P*. *aeruginosa* is a pathogen of high clinical relevance that has acquired resistance to commonly used antibiotics, and is now a priority pathogen on the Centers for Disease Control and Prevention ESKAPE pathogen list (a set of bacteria including *Enterococcus faecium*, *Staphylococcus aureus*, *Klebsiella pneumoniae*, *Acinetobacter baumannii*, *Pseudomonas aeruginosa* and *Enterobacter* spp. designated as multi-drug resistant pathogens requiring new antimicrobials for treatment) [73–75] and a critical pathogen on the World Health Organization’s priority list [76]. Furthermore, it is now well recognized that *lasR* mutants arise during adaptation of *P*. *aeruginosa* to the cystic fibrosis lung environment [34–36]. The LasI/R system is at the top of the QS hierarchy and LasR:3OC12-HSL is required for *rhlI* expression, activating *rhlI* transcription 20-fold (Fig 1, [17]). Thus, a longstanding mystery of urgent clinical importance has been to understand how QS-regulated virulence factors continue to be expressed in the *lasR* mutants obtained from patients. Our work provides insight into one possible mechanism: the alternative RhlR ligand stabilizes the basal level of RhlR protein that is produced in the absence of LasR, enabling RhlR-dependent virulence gene expression. We propose that targeting RhlR with small molecule inhibitors could provide an exciting path forward for the development of novel antimicrobials.

## Materials and methods

### Strains and growth conditions

*P*. *aeruginosa* UCBPP-PA14 strain was grown in lysogeny broth (LB) (10 g tryptone, 5 g yeast extract, 5 g NaCl per L), in 1% Tryptone broth (TB) (10 g tryptone per L) and on LB plates fortified with 1.5% Bacto agar at 37°C. When appropriate, antimicrobials were included at the following concentrations: 400 μg/mL carbenicillin, 30 μg/mL gentamycin, 100 μg/mL irgasan, 750 μg/mL trimethoprim.

### Strain construction

To construct marker-less in-frame chromosomal deletions in *P*. *aeruginosa*, DNA fragments flanking the gene of interest were amplified, assembled by the Gibson method, and cloned into pEXG2 (a generous gift from Dr. Joseph Mougous) [77,78]. The resulting plasmids were used to transform *Escherichia coli* SM10λ*pir*, and subsequently, mobilized into *P*. *aeruginosa* PA14 via biparental mating. Exconjugants were selected on LB containing gentamicin and irgasan, followed by recovery of deletion mutants on LB medium containing 5% sucrose. Candidate mutants were confirmed by PCR. The *rhlR*^*W11STOP*^, *rhlI*^*F50STOP*^ and *rhlA*^*C11STOP*^ mutants were generated by the above method using overlapping Gibson assembly primers containing the mutations. The *rhlR* complementation plasmid was constructed by inserting DNA containing ~500 bp upstream of the *rhlR* gene and the entire *rhlR* open-reading frame using HindIII and SalI, followed by cloning into similarly digested pUCP18 [79].

To construct the P*rhlA*-*mNeonGreen* transcriptional reporter fusion, 500 bp of DNA upstream of the *rhlA* gene and the DNA encoding the *mNeonGreen* open-reading frame were amplified using *P*. *aeruginosa* PA14 genomic DNA and the plasmid p*mNeonGreen*-N1 (licensed from Allele Biotech) as templates, respectively [80]. Next, two DNA fragments of ~730 bp, one corresponding to the intergenic region ~700 bp downstream of the *P*. *aeruginosa PA14_20500* gene and the other corresponding to ~1000 bp upstream of the *P*. *aeruginosa PA14_20510* gene, were amplified from *P*. *aeruginosa* PA14 genomic DNA. The four DNA fragments were assembled by the Gibson method and cloned into pEXG2. The resulting plasmid was used to transform *E*. *coli* SM10λ*pir*, and subsequently mobilized into *P*. *aeruginosa* PA14 WT and the Δ*rhlR* and Δ*rhlI* mutants via biparental mating as described above. The P*chiC*-*mNeonGreen* transcriptional reporter fusion was generated analogously to the P*rhlA*-*mNeonGreen* reporter fusion and integrated on the chromosome at the identical ectopic locus.

The L-arabinose inducible P_BAD_-*rhlR* construct was generated by inserting the DNA encoding the RhlR open reading frame between the NcoI and EcoRI sites on the pTJ1 plasmid [81]. The construct was mobilized into the Δ*lasR* Δ*lasI* Δ*rhlR* Δ*rhlI* quadruple mutant (called Δ4) as described previously [78]. Next, the P*rhlA-mNeonGreen* transcriptional reporter plasmid was conjugated into the Δ4 P_BAD_-*rhlR* strain as described above. To construct constitutively bioluminescent strains for mouse infections, the plasmid pUC18T-miniTn*7*T-*lux*-Tp was mobilized into the WT, Δ*lasR*, Δ*lasI*, Δ*rhlR*, and Δ*rhlI* mutants as described previously [82,83]. The strains and plasmids used in this study are listed in S3 Table.

### Pyocyanin assay

*P*. *aeruginosa* strains were grown overnight in LB liquid medium at 37°C with shaking. Cultures were back diluted 1:1000 into fresh medium and grown for 18 h. The cells were pelleted by centrifugation, and the culture fluids were passed through 0.22 μm filters into clear plastic cuvettes. The OD_695_ of each sample was measured on a spectrophotometer (Beckman Coulter DV 730).

To quantify pyocyanin production from colony biofilms, 10 μL of culture was spotted onto 60 x 15 mm Petri plates containing 10 mL 1% Tryptone medium solidified with 1% agar. The plates were incubated at 25°C for 5 days. Pyocyanin was extracted by the addition of 5 ml chloroform to the plate, followed by a second extraction with 0.2 N HCl. The absorbance of this solution was measured on a spectrophotometer at 530 nm (Beckman Coulter DV 730). The OD_520_ of each sample was multiplied by 17.072 to obtain the concentration of pyocyanin (μg per CFU) [84].

### Colony biofilm assay

One microliter of overnight *P*. *aeruginosa* cultures grown at 37°C in 1% Tryptone broth was spotted onto 60 x 15 mm Petri plates containing 10 mL 1% Tryptone medium fortified with 40 mg per L Congo red and 20 mg per L Coomassie brilliant blue dyes, and solidified with 1% agar. Colonies were grown at 25°C and images were acquired after 120 h using a Leica stereomicroscope M125 mounted with a Leica MC170 HD camera at 7.78x zoom.

### RNA-seq

*P*. *aeruginosa* strains were harvested from HCD planktonic cultures (OD_600_ = 2.0) and from mature (5-day old) colony biofilms. RNA was purified using Trizol (Ambion). 0.5 μg of total RNA was subjected to rRNA-depletion using the Ribo-Zero rRNA Removal Kit (Bacteria) from Illumina, followed by library preparation using the PrepX RNA-Seq Illumina Library Kit (WaferGen Biosystems). Unique barcodes were added to each sample pool to enable multiplexing. Libraries were sequenced as single end 75 bp reads on an Illumina HiSeq 2500 instrument. Data analysis was performed on a local Galaxy platform. Reads (~10 million reads per replicate) were mapped to the *P*. *aeruginosa* UCBPP-PA14 genome (www.pseudomonas.com, [85]) using TopHat. Differentially expressed genes were identified using DESeq2. Genes showing log_2_ fold-change > or = 2 and adjusted P-value < or = 0.001 in the mutant strains compared to WT, under the corresponding condition, are reported in this study.

### qRT-PCR

*P*. *aeruginosa* strains were harvested from HCD planktonic cultures (OD_600_ = 2.0) and from mature (5-day old) colony biofilms. RNA was purified using Trizol (Ambion), and subsequently, DNAse treated (TURBO DNA-free, Thermo Fisher). cDNA was synthesized using SuperScript III Reverse Transcriptase (Invitrogen) and quantified using PerfeCTa SYBR Green FastMix Low ROX (Quanta BioSciences).

### *rhlA* reporter assay

WT and mutant *P*. *aeruginosa* strains harboring the chromosomally encoded P*rhlA*-*mNeonGreen* fusion were grown overnight at 37°C. The cultures were diluted 1:1000 in 3 mL of TB medium. When required, DMSO solvent or 20% (v/v) cell-free culture fluids were added and the cultures were incubated at 37°C until the OD_600_ reached 2.0. 1 mL of culture was harvested, the supernatant was removed, and the cells were resuspended in PBS. 200 μL of culture suspension was transferred to wells of 96 well plates and fluorescence was measured using an Envision 2103 Multilabel Reader (Perkin Elmer) using the FITC filter with an excitation of 485 nM and emission of 535 nM.

### *C*. *elegans* fast killing assay

WT N2 worms were propagated on *E*. *coli* OP50 lawns on Nematode Growth Media (NGM) plates [86]. Gravid adults were allowed to lay eggs on lawns of fresh *E*. *coli* OP50 after which the adults were removed and the eggs were allowed to grow for 48 h (to reach the L4 stage) at 20°C prior to transfer to lawns of *P*. *aeruginosa* strains at 25°C on PGS plates (1% Bacto-Peptone, 1% NaCl, 1% glucose, 0.15M sorbitol, 1.7% Bacto-Agar). Nematodes were scored for survival at 4, 16, and 24 h time points (30 worms per replicate, three replicates performed). Data were plotted and SEM determined using GraphPad Prism software.

### Murine infection assays

*P*. *aeruginosa* strains were grown on *Pseudomonas* Isolation Agar (PIA) for 16–18 h at 37°C and suspended in PBS to an OD_600_ of 0.5, corresponding to ~10^9^ CFU/mL. Inocula were adjusted spectrophotometrically to obtain the desired challenge dose in a volume of 50 μL. Six-week old female Balb/c mice (Jackson Laboratories, Bar Harbor, ME) were anesthetized by i.p. injection of 0.2 mL of a mixture of ketamine (25 mg/mL) and xylaxine (12 mg/mL). Mice were infected by non-invasive intratracheal instillation of dilutions of *P*. *aeruginosa* PA14 *P1-lux* or isogenic QS mutants as previously described [87]. Mice were observed over 5 days, and animals that succumbed to infection or appeared to be under acute distress were humanely euthanized and were included in the experiment results. To determine each LD_50_, groups of mice were challenged with different doses of *P*. *aeruginosa* PA14 or isogenic QS mutants. Four mice were tested with each dose of each strain. The percent lethality corresponding to each dose was assessed. The LD_50_ was calculated using the method of Reed and Muench [88]. Data were analyzed by the log-rank test. All survival experiments were repeated at least three times.

For colonization of mice, *P*. *aeruginosa* strains were grown and prepared as described above. Six-week-old female Balb/c mice were anesthetized and infected with sublethal doses (~0.5 LD_50_) of *P*. *aeruginosa* PA14 *P1-lux* or isogenic QS mutants as described in the preceding section. Mice were euthanized at 24 and 48 h post-infection and whole lungs were collected aseptically, weighed, and homogenized in 1 mL of PBS. Tissue homogenates were serially diluted and plated on PIA and CFU determination was made 16–18 h later. Comparison of the numbers of viable bacteria obtained in lung homogenates relied on the Kruskal-Wallis test for three group analyses or the Mann-Whitney *U* test for two group analyses.

An additional group of animals was included for each *P*. *aeruginosa* strain examined for real time monitoring of colonization and localization of bioluminescent bacteria using an IVIS–Lumina LT III imaging system (PerkinElmer). Briefly, each group of mice was anesthetized with 3% isoflurane using an XGI-8 Gas Anesthesia System (Caliper Life Sciences), and imaged using medium binning, f/stop 1, subject height 1.5 cm. Images were acquired with up to 5 min exposure. Total photon emission from the ventral and dorsal sides of imaged mice was quantified using Living Image Software v4.0x (Xenogen Corp.). Due to the differences in the virulence level and resulting bacterial load, mice infected with *P*. *aeruginosa* PA14 *P1-lux* or isogenic QS mutants were monitored separately, but individual mice were compared at 24 and 48 h under the same conditions. All correlations were done as average radiance of photons emitted per second, area, and steradian (p/s/cm^2^/sr) under the chosen experimental conditions. All colonization and imaging experiments were repeated at least twice. Statistical analyses were performed using GraphPad Prism software.

### LC-MS for detection of C4-HSL

Samples were diluted 1:1 with methanol (MeOH). Synthetic C4-HSL (Sigma-Aldrich) standards were prepared in 50% MeOH. Samples and standards were loaded onto a 1 mm x 150 mm C12 column (Phenomenex, Jupitor 4 μm Proteo 90A) using a Shimadzu HPLC system and PAL auto-sampler (20 μL/injection) at a flowrate of 70 μL/min. The column was maintained at 35°C using a column oven. The column was connected inline to an electrospray source coupled to an LTQ-Orbitrap XL mass spectrometer (ThermoFisher). Caffeine (5 pM/μL in 50% Acetonitrile with 0.1% Formic Acid) was injected as a lock mass through a tee at the column outlet using a syringe pump at 10 μL/min (Harvard PHD 2000). Chromatographic separation was achieved with a linear gradient from 1% to 99% B in A in 8.5 min (A: 0.1% Formic Acid, B: 0.1% Formic Acid in Acetonitrile) with an initial 1 min hold at 1% B and followed by 5 min wash at 100% B and equilibration for 10 min with 1% B (total program was 20 min). Electrospray ionization was achieved using a spray voltage of 4.5 kV aided by sheath gas (Nitrogen) flow rate of 12 (arbitrary units) and auxiliary gas (Nitrogen) flow rate of 1 (arbitrary units). Full scan MS data were acquired in the Orbitrap at a resolution of 60,000 in profile mode from the m/z range of 160–320. LC-MS data were manually interpreted using the Xcalibur Qual browser (Thermo, Version 2.1) to visualize C4-HSL mass spectra and to generate extracted ion chromatograms using the theoretical [M+H] within a range of ±2 ppm.

### Ethics statement

All animal procedures were conducted according to the guidelines of the Emory University Institutional Animal Care and Use Committee (IACUC), under approved protocol number DAR-2003421-042219BN. The study was carried out in strict accordance with established guidelines and policies at Emory University School of Medicine, and recommendations in the Guide for Care and Use of Laboratory Animals of the National Institute of Health, as well as local, state, and federal laws.

## Supporting information

S1 Fig The *P*. *aeruginosa* colony biofilm morphology develops over time and is independent of Congo red in the assay medium. A) Five-day time courses showing development of the colony biofilm morphologies of the WT, Δ*rhlR* mutant, and Δ*rhlI* mutant. B) Colony biofilm morphologies of the WT, Δ*rhlR* mutant transformed with the empty pUCP18 plasmid, and the Δ*rhlR* Δ*rhlI* double mutant transformed with *rhlR* on the pUCP18 plasmid under its native promoter. C) Colony biofilm morphologies of the strains in panel A after 5 days on medium lacking the Congo red and Coomassie brilliant blue dyes. Scale bar is 2 mm.
(TIF)

S2 Fig *P*. *aeruginosa* Δ*lasR* and Δ*lasI* mutants have identical phenotypes whereas the *rhlR*^*W10STOP*^ and *rhlI*^*F50STOP*^ mutants do not. A) Pyocyanin production (OD_695_) in cell-free culture fluids prepared from the WT, Δ*lasR*, Δ*lasI*, *rhlR*^*W10STOP*^, and *rhlI*^*F50STOP*^ mutants. Error bars represent SD for three biological replicates. B) Colony biofilm phenotypes of the WT, Δ*lasR* and Δ*lasI* mutants. Scale bar is 2 mm. C) Colony biofilm surface area quantitation of the indicated strains over the course of 5 days. Error bars represent SEM of three independent experiments. D) Colony biofilm morphologies of the WT, *rhlR*^*W10STOP*^ and *rhlI*^*F50STOP*^ mutants. Scale bar is 2 mm.
(TIF)

S3 Fig Rhamnolipids are not responsible for the distinct *P*. *aeruginosa* Δ*rhlR* and Δ*rhlI* colony biofilm phenotypes. Colony biofilm morphology of the WT and *rhlA*^*C11STOP*^, Δ*rhlR rhlA*^*C11STOP*^, and Δ*rhlI rhlA*^*C11STOP*^ mutants. Scale bar is 2 mm.
(TIF)

S4 Fig Pyocyanin levels are elevated in Δ*rhlI P*. *aeruginosa* colony biofilms. Pyocyanin levels were measured in micrograms per colony forming unit for the WT, the Δ*rhlR* and Δ*rhlI* single mutants, and the Δ*rhlI* Δ*phz* double mutant. Error bars represent SEM of three independent replicates.
(TIF)

S5 Fig The Δ*rhlR* mutant colony biofilm morphology requires the Pel exopolysaccharide. Colony biofilm morphology of the WT and the Δ*pelA*, Δ*rhlR* Δ*pelA*, and Δ*rhlI* Δ*pelA* mutants. Scale bar is 2 mm.
(TIF)

S6 Fig Reporter activity for RhlR-controlled class I and class II genes. A) RhlR-directed transcription of a representative Class I gene was measured using an *mNeonGreen* transcriptional reporter fusion to the *chiC* promoter integrated at an ectopic locus on the chromosome. *PchiC-mNeonGreen* reporter activity from the WT grown to HCD in planktonic culture is set to 100%, and reporter activity is shown for the Δ*rhlR* mutant, Δ*rhlI* mutant, Δ*rhlR* mutant complemented with the *rhlR* gene under its native promoter on pUCP18 (p*rhlR*), and the Δ*rhlI* mutant supplied with exogenous 10 μM C4-HSL. Error bars represent SD for three biological replicates. B) As in A for RhlR-directed transcription of the representative Class II gene *rhlA*. Pyocyanin production and biofilm formation are used as readouts for Class III gene behavior and are shown in Fig 2A and 2B respectively, of the main text.
(TIF)

S7 Fig *PrhlA-mNeonGreen* transcriptional reporter activity in WT, QS, and phenazine mutants. A) RhlR-directed transcription was measured using an *mNeonGreen* transcriptional reporter fusion to the *rhlA* promoter integrated at an ectopic locus on the chromosome. *PrhlA-mNeonGreen* reporter activity from the WT grown to HCD in liquid culture is set to 100%, and reporter activity in the Δ*rhlR*, Δ*rhlI*, Δ*lasR*, Δ*lasI*, Δ4 (Δ*rhlR* Δ*rhlI* Δ*lasR* Δ*lasI* quadruple mutant), and Δ4 *P*_*BAD*_*-rhlR* strains are shown. Error bars represent SEM of three independent replicates. B) Gray bars represent *rhlA* reporter activity when *rhlR* was induced in the Δ4 P_BAD_-*rhlR* strain with 0.1% L-arabinose in the presence of 1% DMSO (solvent control) or 10 μM C4-HSL. The *rhlA* reporter activity was set to 100% when 10 μM C4-HSL was added. In the cultures represented by the black bars, P*rhlA-mNeonGreen* was monitored in response to 20% (v/v) of the cell-free culture fluids prepared from the indicated strains. Error bars represent SEM for three biological replicates.
(TIF)

S8 Fig Cell-free culture fluids from the Δ*rhlI* mutant lack C4-HSL. The total ion currents measured within 2 ppm of the predicted mass of C4-HSL are shown across the liquid chromatography gradient for 500 nM C4-HSL (top, black line), and from cell-free culture fluids prepared from 1 mL of HCD planktonic cultures of WT *P*. *aeruginosa* PA14 (middle, red line) and the Δ*rhlI* mutant (bottom, green line). Peaks above the 10% signal-to-noise threshold are labeled with retention times (RT) and peak areas (MA). The Y-axes show the normalized values with 4 X 10^5^ arbitrary units set as 100% in each panel.
(TIF)

S9 Fig The Δ*lasI* and Δ*lasR* mutants exhibit different phenotypes in nematode and murine infection models. A) *C*. *elegans* were applied to lawns of *E*. *coli* OP50 (open squares), WT *P*. *aeruginosa* PA14 (closed diamonds), Δ*lasR* mutant (closed triangles), and Δ*lasI* mutant (closed circles). Error bars represent SEM of three independent replicates. B) Real-time monitoring of WT *P*. *aeruginosa* PA14 *P1-lux* and isogenic mutants in the acute pneumonia model. BALB/c mice infected intratracheally with WT, Δ*lasR*, and Δ*lasI* strains were imaged at 24 and 48 h using an IVIS CCD camera. Imaging was performed from the ventral side of representative mice while the animals were under isoflourane anesthesia. The color bars indicate the intensity of the bioluminescence output, with red and blue denoting the high and low signals, respectively. Note that the color scales on the various mouse bioluminescence imaging panels are not the same. (C) Bacterial load in lung homogenates of mice infected intratracheally with WT *P*. *aeruginosa* PA14 and the Δ*lasR* and Δ*lasI* mutants. Each symbol represents a single mouse. The data are pooled from two independent experiments. Data were analyzed using the Mann-Whitney U test. *** P <0.001 and ns means not significant.
(TIF)

S10 Fig Phenazines are required for virulence in the nematode infection model. *C*. *elegans* were applied to lawns of WT *P*. *aeruginosa* PA14 (closed diamonds), the Δ*rhlI* mutant (closed circles), the Δ*phz* mutant (open squares), and the Δ*rhlI* Δ*phz* mutant (open circles). Error bars represent SEM of three independent replicates.
(TIF)

S11 Fig Pyocyanin production by *P*. *aeruginosa* PA14 WT and QS mutant strains. A-E) WT, Δ*rhlR*, Δ*rhlI*, Δ*lasR*, and Δ*lasI P*. *aeruginosa* PA14 lawns, and F) *E*. *coli* OP50 lawn on PGS agar plates.
(TIF)

S1 Table Genes controlled in *P*. *aeruginosa* PA14 by RhlR and RhlI in HCD planktonic culture determined by RNA-seq. (DOCX)

S2 Table Genes controlled in *P*. *aeruginosa* PA14 by RhlR and RhlI in colony biofilms determined by RNA-seq. (DOCX)

S3 Table Bacterial strains and plasmids. (DOCX)
