# Supplementary figures and images for: The RhlR quorum-sensing receptor controls Pseudomonas aeruginosa pathogenesis and biofilm development independently of its canonical homoserine lactone autoinducer
Source: PLoS Pathog. 2017 Jul 17;13(7):e1006504. doi: 10.1371/journal.ppat.1006504 (PMC5531660; doi:10.1371/journal.ppat.1006504)

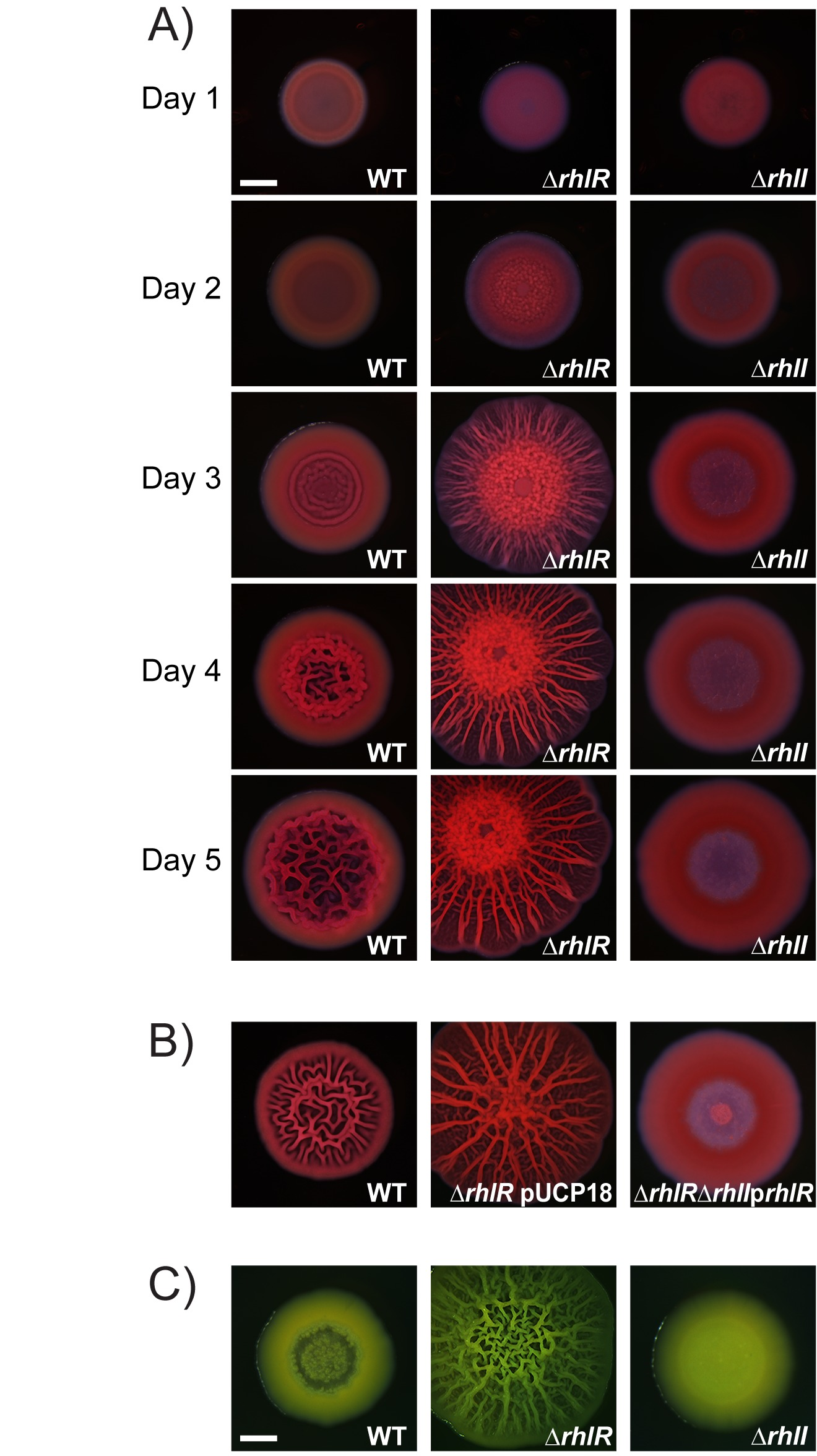

Supplement: S1 Fig — A) Five-day time courses showing development of the colony biofilm morphologies of the WT, ΔrhlR mutant, and ΔrhlI mutant. B) Colony biofilm morphologies of the WT, ΔrhlR mutant transformed with the empty pUCP18 plasmid, and the ΔrhlR ΔrhlI double mutant transformed with rhlR on the pUCP18 plasmid under its native promoter. C) Colony biofilm morphologies of the strains in panel A after 5 days on medium lacking the Congo red and Coomassie brilliant blue dyes. Scale bar is 2 mm. (TIF) [file ppat.1006504.s001.tif]

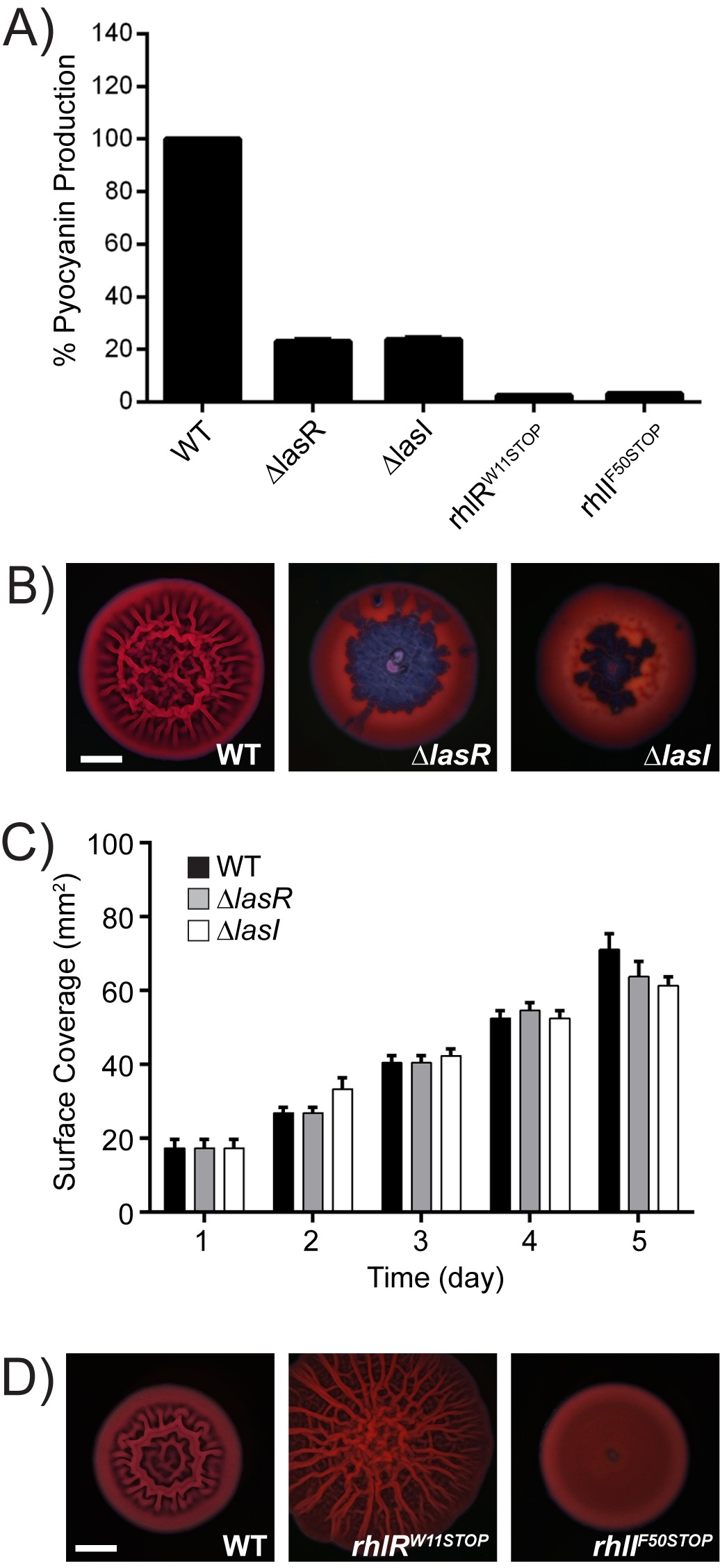

Supplement: S2 Fig — A) Pyocyanin production (OD695) in cell-free culture fluids prepared from the WT, ΔlasR, ΔlasI, rhlRW10STOP, and rhlIF50STOP mutants. Error bars represent SD for three biological replicates. B) Colony biofilm phenotypes of the WT, ΔlasR and ΔlasI mutants. Scale bar is 2 mm. C) Colony biofilm surface area quantitation of the indicated strains over the course of 5 days. Error bars represent SEM of three independent experiments. D) Colony biofilm morphologies of the WT, rhlRW10STOP and rhlIF50STOP mutants. Scale bar is 2 mm. (TIF) [file ppat.1006504.s002.tif]

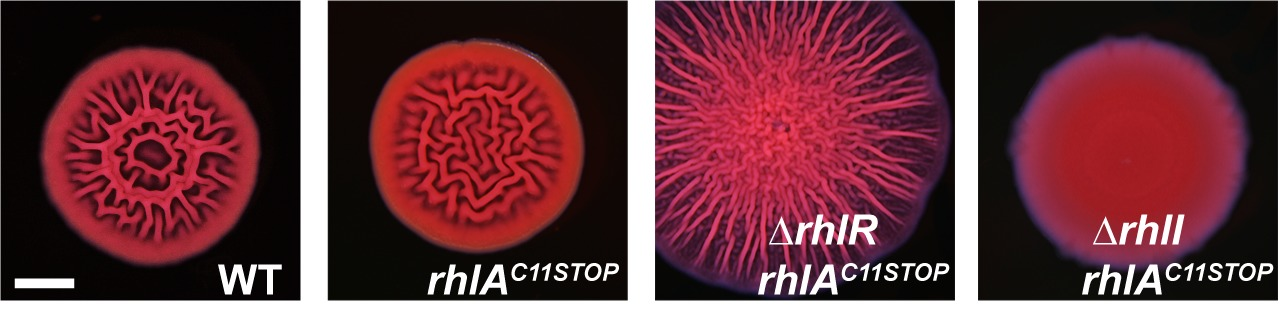

Supplement: S3 Fig — Colony biofilm morphology of the WT and rhlAC11STOP, ΔrhlR rhlAC11STOP, and ΔrhlI rhlAC11STOP mutants. Scale bar is 2 mm. (TIF) [file ppat.1006504.s003.tif]

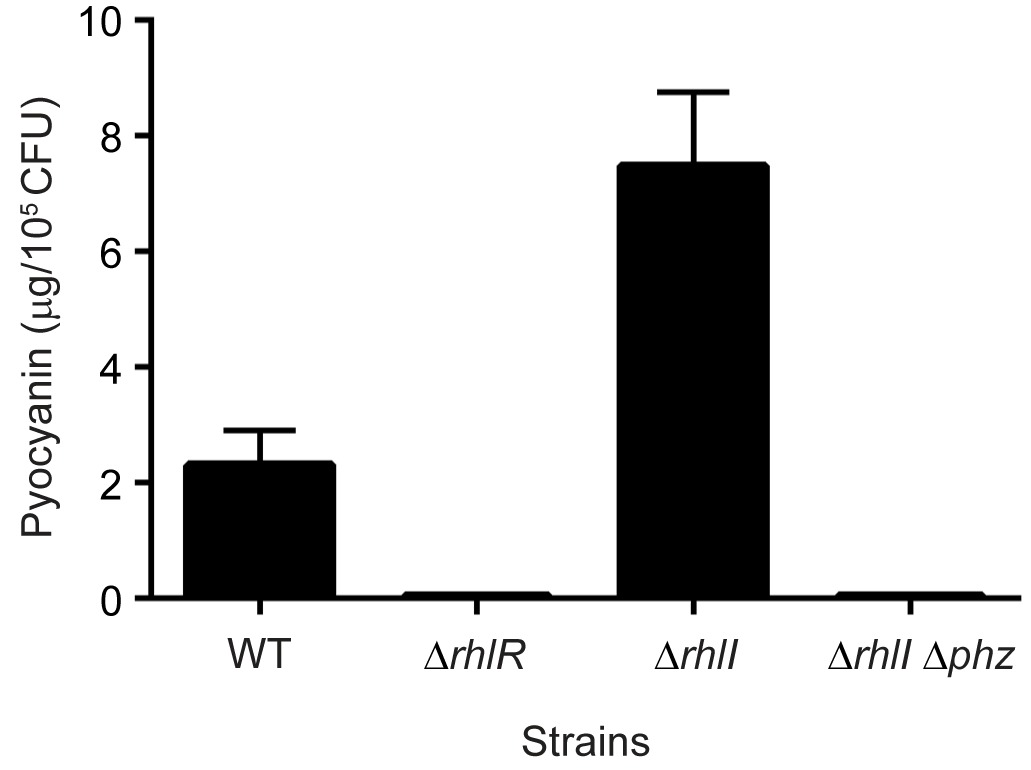

Supplement: S4 Fig — Pyocyanin levels were measured in micrograms per colony forming unit for the WT, the ΔrhlR and ΔrhlI single mutants, and the ΔrhlI Δphz double mutant. Error bars represent SEM of three independent replicates. (TIF) [file ppat.1006504.s004.tif]

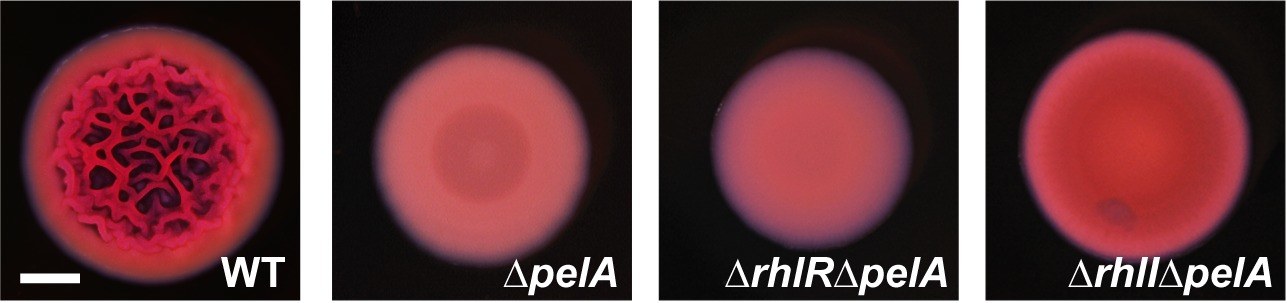

Supplement: S5 Fig — Colony biofilm morphology of the WT and the ΔpelA, ΔrhlR ΔpelA, and ΔrhlI ΔpelA mutants. Scale bar is 2 mm. (TIF) [file ppat.1006504.s005.tif]

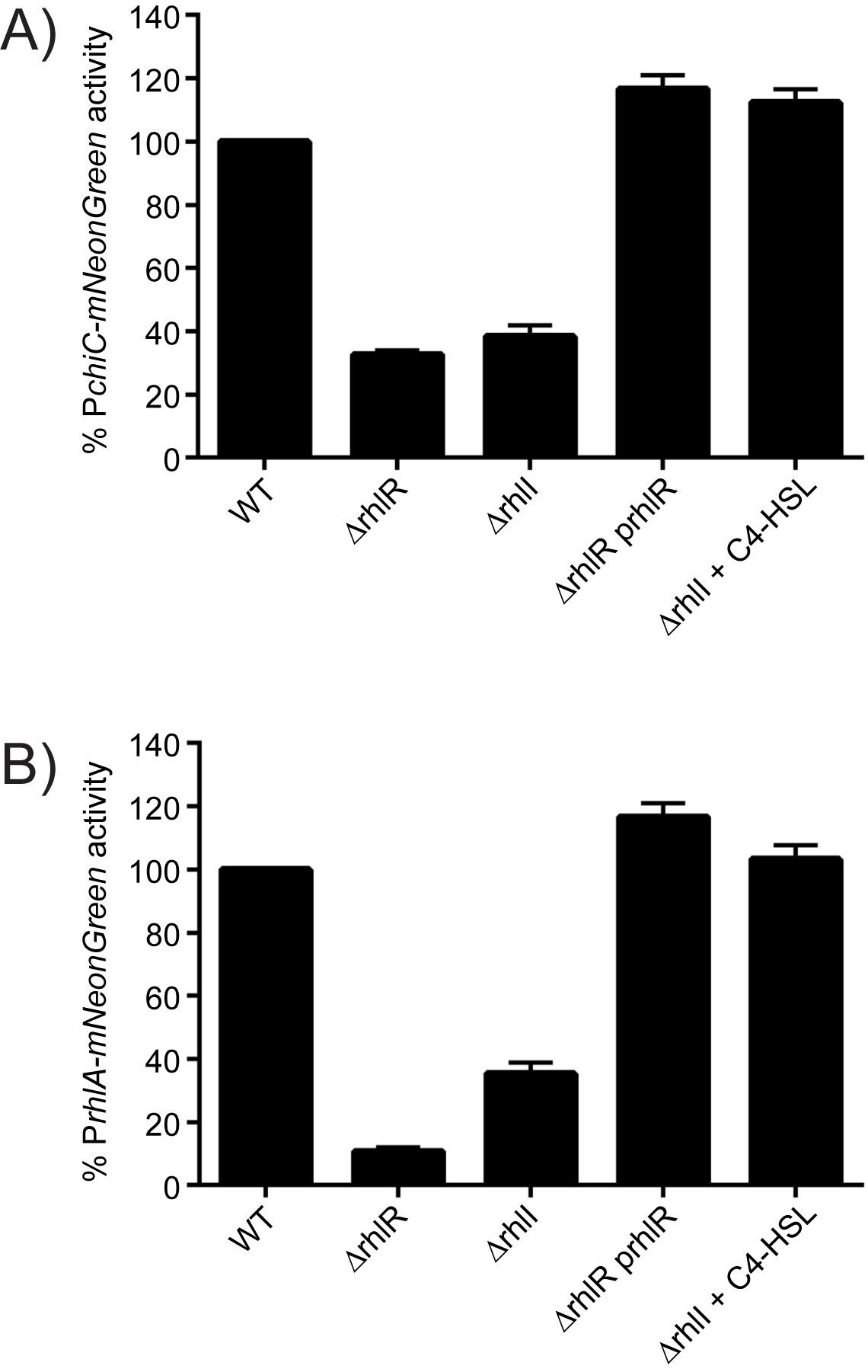

Supplement: S6 Fig — A) RhlR-directed transcription of a representative Class I gene was measured using an mNeonGreen transcriptional reporter fusion to the chiC promoter integrated at an ectopic locus on the chromosome. PchiC-mNeonGreen reporter activity from the WT grown to HCD in planktonic culture is set to 100%, and reporter activity is shown for the ΔrhlR mutant, ΔrhlI mutant, ΔrhlR mutant complemented with the rhlR gene under its native promoter on pUCP18 (prhlR), and the ΔrhlI mutant supplied with exogenous 10 μM C4-HSL. Error bars represent SD for three biological replicates. B) As in A for RhlR-directed transcription of the representative Class II gene rhlA. Pyocyanin production and biofilm formation are used as readouts for Class III gene behavior and are shown in Fig 2A and 2B respectively, of the main text. (TIF) [file ppat.1006504.s006.tif]

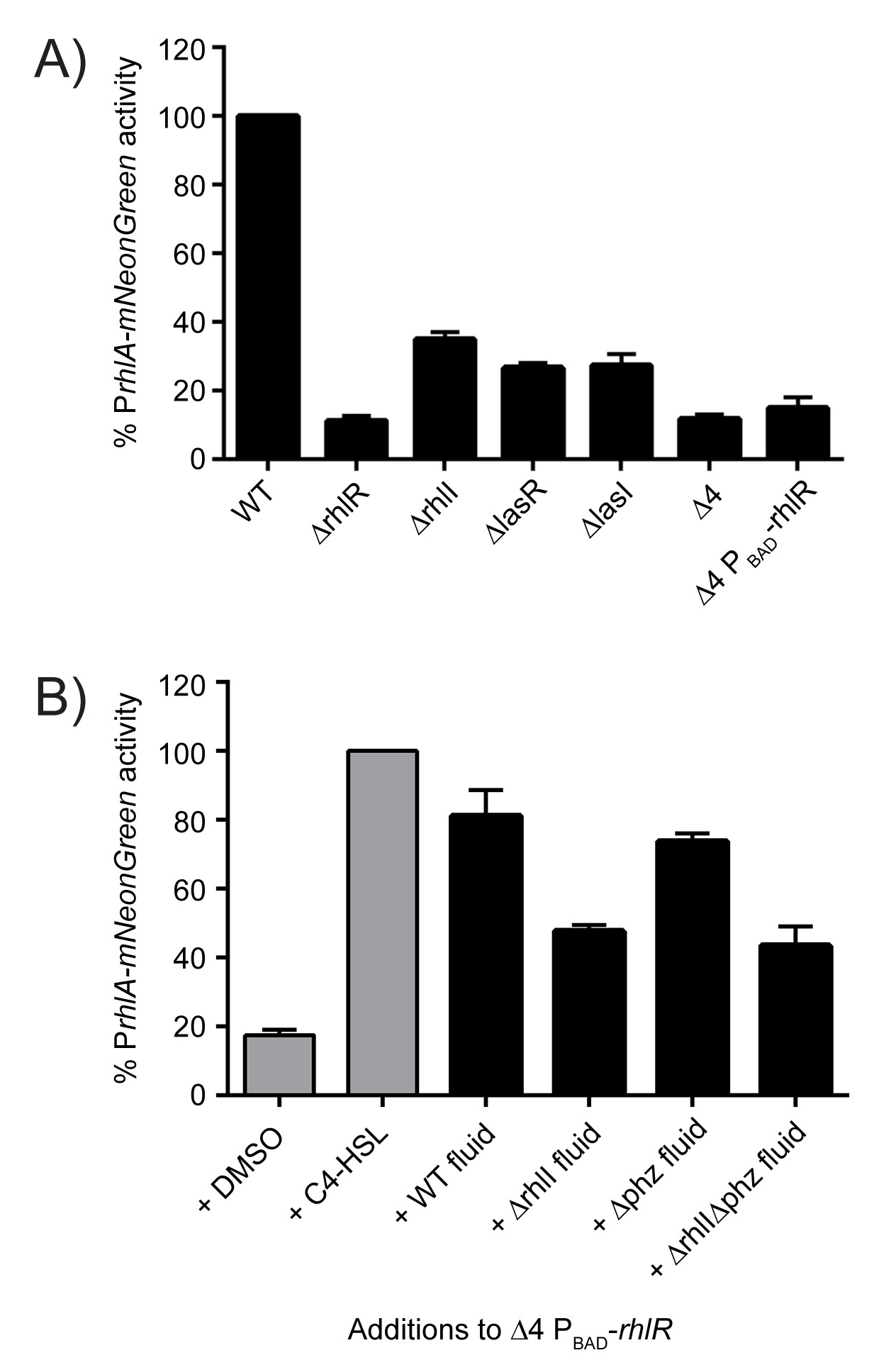

Supplement: S7 Fig — A) RhlR-directed transcription was measured using an mNeonGreen transcriptional reporter fusion to the rhlA promoter integrated at an ectopic locus on the chromosome. PrhlA-mNeonGreen reporter activity from the WT grown to HCD in liquid culture is set to 100%, and reporter activity in the ΔrhlR, ΔrhlI, ΔlasR, ΔlasI, Δ4 (ΔrhlR ΔrhlI ΔlasR ΔlasI quadruple mutant), and Δ4 PBAD-rhlR strains are shown. Error bars represent SEM of three independent replicates. B) Gray bars represent rhlA reporter activity when rhlR was induced in the Δ4 PBAD-rhlR strain with 0.1% L-arabinose in the presence of 1% DMSO (solvent control) or 10 μM C4-HSL. The rhlA reporter activity was set to 100% when 10 μM C4-HSL was added. In the cultures represented by the black bars, PrhlA-mNeonGreen was monitored in response to 20% (v/v) of the cell-free culture fluids prepared from the indicated strains. Error bars represent SEM for three biological replicates. (TIF) [file ppat.1006504.s007.tif]

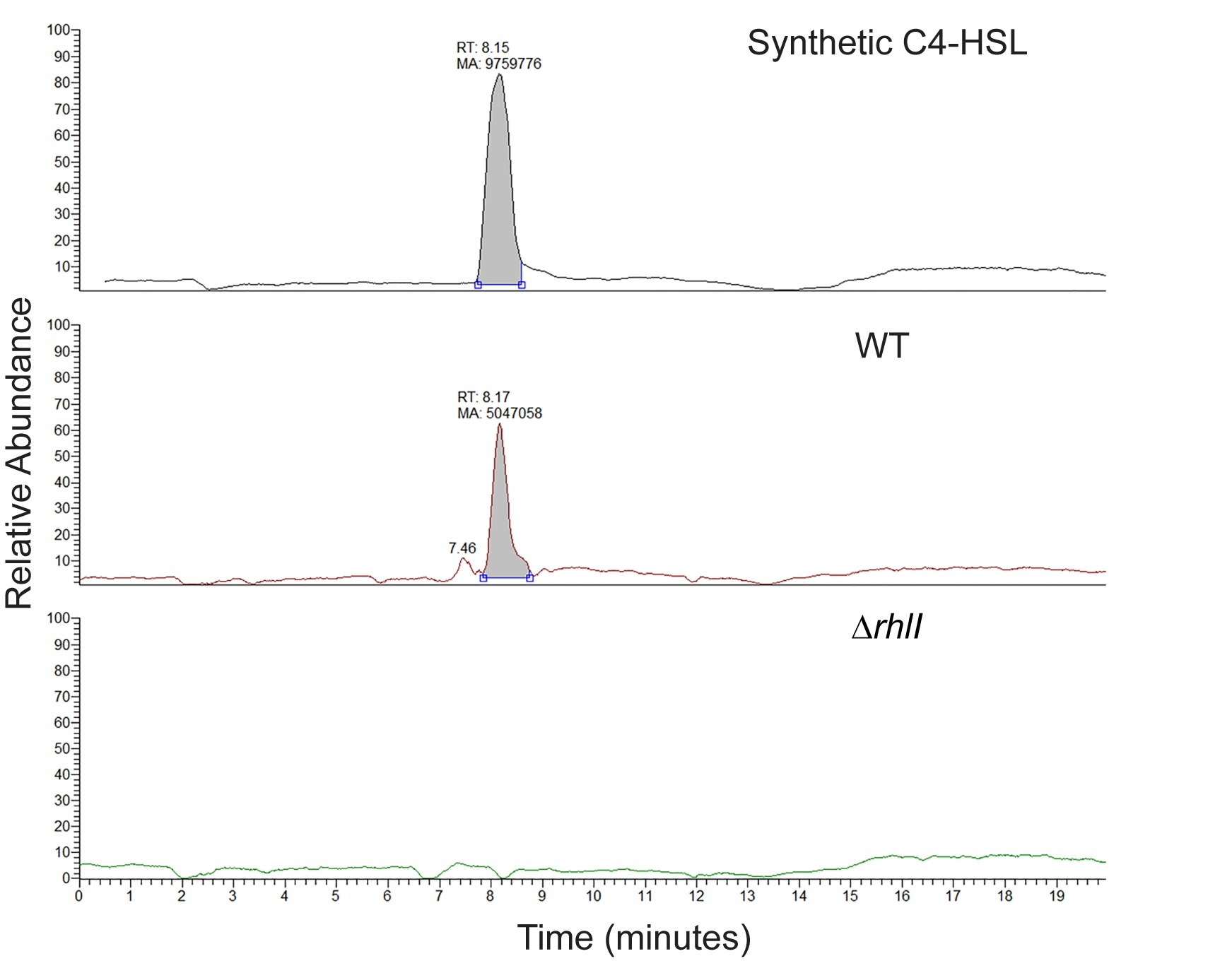

Supplement: S8 Fig — The total ion currents measured within 2 ppm of the predicted mass of C4-HSL are shown across the liquid chromatography gradient for 500 nM C4-HSL (top, black line), and from cell-free culture fluids prepared from 1 mL of HCD planktonic cultures of WT P. aeruginosa PA14 (middle, red line) and the ΔrhlI mutant (bottom, green line). Peaks above the 10% signal-to-noise threshold are labeled with retention times (RT) and peak areas (MA). The Y-axes show the normalized values with 4 X 105 arbitrary units set as 100% in each panel. (TIF) [file ppat.1006504.s008.tif]

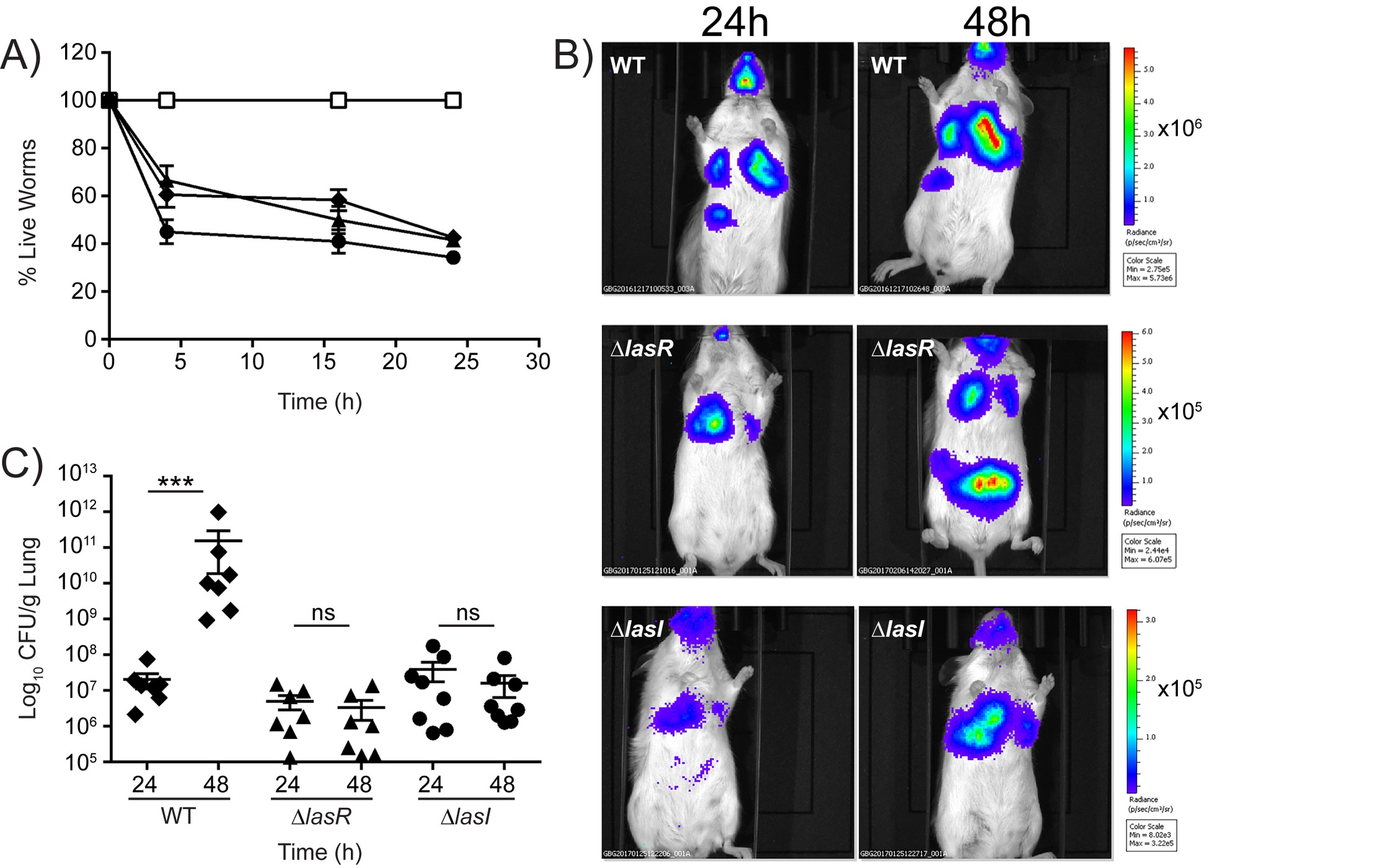

Supplement: S9 Fig — A) C. elegans were applied to lawns of E. coli OP50 (open squares), WT P. aeruginosa PA14 (closed diamonds), ΔlasR mutant (closed triangles), and ΔlasI mutant (closed circles). Error bars represent SEM of three independent replicates. B) Real-time monitoring of WT P. aeruginosa PA14 P1-lux and isogenic mutants in the acute pneumonia model. BALB/c mice infected intratracheally with WT, ΔlasR, and ΔlasI strains were imaged at 24 and 48 h using an IVIS CCD camera. Imaging was performed from the ventral side of representative mice while the animals were under isoflourane anesthesia. The color bars indicate the intensity of the bioluminescence output, with red and blue denoting the high and low signals, respectively. Note that the color scales on the various mouse bioluminescence imaging panels are not the same. (C) Bacterial load in lung homogenates of mice infected intratracheally with WT P. aeruginosa PA14 and the ΔlasR and ΔlasI mutants. Each symbol represents a single mouse. The data are pooled from two independent experiments. Data were analyzed using the Mann-Whitney U test. *** P <0.001 and ns means not significant. (TIF) [file ppat.1006504.s009.tif]

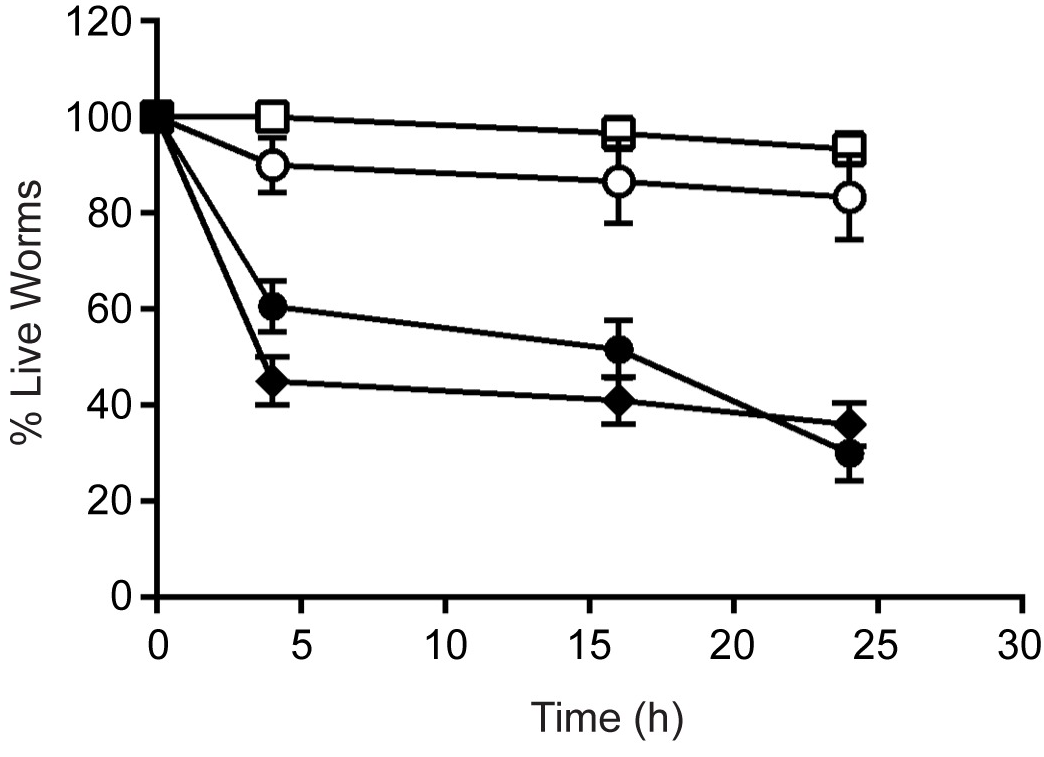

Supplement: S10 Fig — C. elegans were applied to lawns of WT P. aeruginosa PA14 (closed diamonds), the ΔrhlI mutant (closed circles), the Δphz mutant (open squares), and the ΔrhlI Δphz mutant (open circles). Error bars represent SEM of three independent replicates. (TIF) [file ppat.1006504.s010.tif]

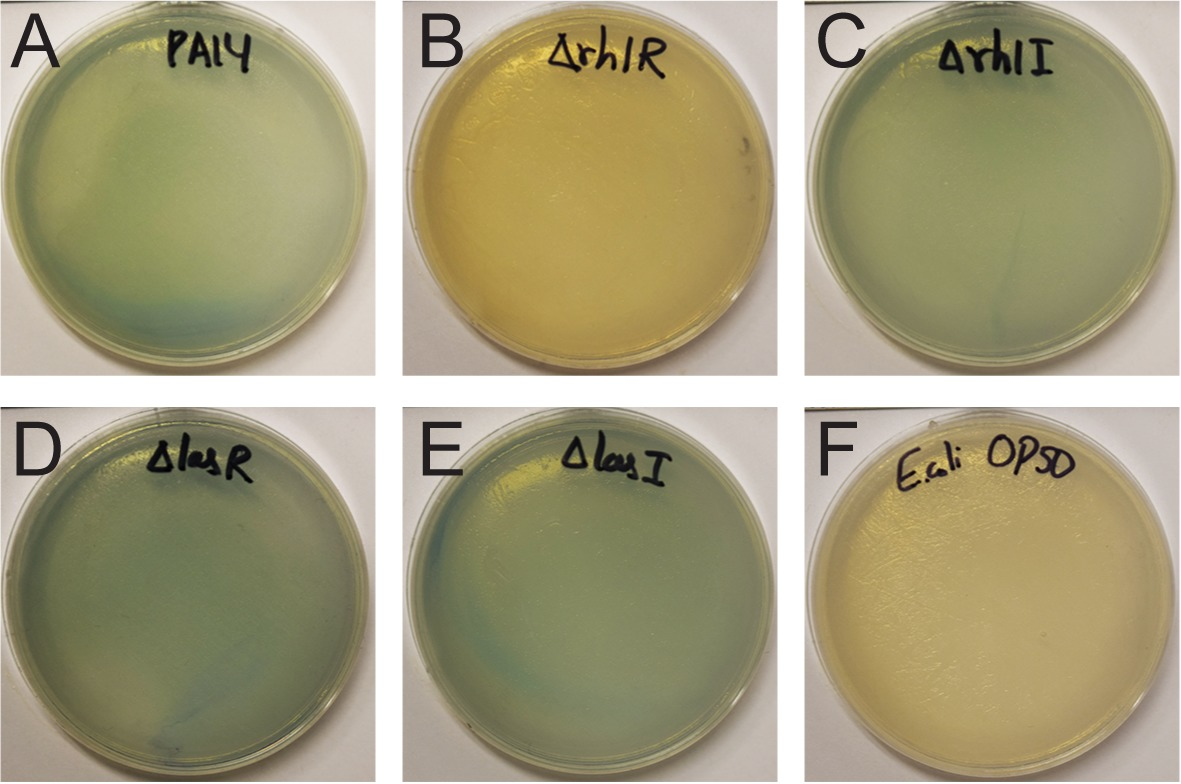

Supplement: S11 Fig — A-E) WT, ΔrhlR, ΔrhlI, ΔlasR, and ΔlasI P. aeruginosa PA14 lawns, and F) E. coli OP50 lawn on PGS agar plates. (TIF) [file ppat.1006504.s011.tif]
