# Supplementary material for: The RhlR quorum-sensing receptor controls Pseudomonas aeruginosa pathogenesis and biofilm development independently of its canonical homoserine lactone autoinducer
Source: PLoS Pathog. 2017 Jul 17;13(7):e1006504. doi: 10.1371/journal.ppat.1006504 (PMC5531660; doi:10.1371/journal.ppat.1006504)
Supplement: S1 Table — (DOCX) [file ppat.1006504.s012.docx]

**S1Table. Genes controlled in *P. aeruginosa* PA14 by RhlR and RhlI in HCD^a^ planktonic culture determined by RNA-seq.**

| **PA14 ID^b,c^** | **log_2_-fold change^d,e^** | | **Gene name and description** |
| --- | --- | --- | --- |
|  | **Δ*rhlR*** | **Δ*rhlI*** |  |
| PA14_00640 | 3.46 | 3.01 | *phzH*, potential phenazine-modifying enzyme |
| PA14_01490 | 3.41 | - | hemolysin |
| PA14_01560 | -1.85 | -1.48 | hypothetical protein |
| PA14_01710 | 1.81 | 2.10 | *ahpC*, alkyl hydroperoxide reductase |
| PA14_01720 | 3.34 | 3.35 | *ahpF*, alkyl hydroperoxide reductase |
| PA14_02910 | 1.77 | - | IclR family transcriptional regulator |
| PA14_03080 | 1.88 | 1.98 | acetyltransferase |
| PA14_03090 | 3.15 | 3.05 | hypothetical protein |
| PA14_03490 | 1.94 | 1.56 | hypothetical protein |
| PA14_03520 | 2.02 | - | hypothetical protein |
| PA14_05660 | 1.18 | - | transcriptional regulator |
| PA14_07070 | -1.49 | -1.20 | hypothetical protein |
| PA14_09400 | 2.19 | - | *phzS*, hypothetical protein |
| PA14_09480 | 2.33 | - | *phzA1*, phenazine biosynthesis protein |
| PA14_09490 | 2.37 | - | *phzM*, phenazine-specific methyltransferase |
| PA14_09500 | 6.50 | 2.49 | *opmD*, outer membrane protein |
| PA14_09520 | 5.66 | 2.41 | *mexI*, RND efflux transporter |
| PA14_09530 | 5.32 | 2.47 | *mexH*, RND efflux membrane fusion protein |
| PA14_09540 | 5.71 | 2.68 | *mexG*, hypothetical protein |
| PA14_10340 | 2.03 | - | toxin transporter |
| PA14_10350 | 3.81 | 2.74 | secretion protein |
| PA14_10360 | 4.91 | 3.06 | hypothetical protein |
| PA14_10380 | - | 1.78 | hypothetical protein |
| PA14_11130 | 2.08 | 1.42 | short chain dehydrogenase |
| PA14_11140 | 3.09 | 1.90 | nonribosomal peptide synthetase |
| PA14_11790 | -1.56 | - | amino acid transporter |
| PA14_13340 | 2.13 | - | extracellular nuclease |
| PA14_14320 | 2.26 | - | hypothetical protein |
| PA14_14360 | -0.89 | - | sodium:sulfate symporter |
| PA14_14710 | 1.73 | 1.43 | Rrf2 family protein |
| PA14_14730 | 1.97 | 1.66 | *iscS*, cysteine desulfurase |
| PA14_14740 | 2.12 | 2.16 | scaffold protein |
| PA14_14750 | 1.92 | 1.76 | iron-binding protein IscA |
| PA14_14770 | 1.85 | 1.14 | *hscB*, co-chaperone HscB |
| PA14_14780 | 1.73 | 1.22 | *hscA*, chaperone protein HscA |
| PA14_15070 | 1.41 | - | *oprC*, outer membrane copper receptor OprC |
| PA14_15130 | 1.28 | - | hypothetical protein |
| PA14_16100 | 2.22 | - | hypothetical protein |
| PA14_16250 | 2.07 | - | *lasB*, elastase LasB |
| PA14_16310 | 3.32 | 2.51 | MFS permease |
| PA14_18070 | -1.62 | -1.71 | periplasmic metal-binding protein |
| PA14_18590 | - | 2.03 | hypothetical protein |
| PA14_18800 | 2.69 | 1.25 | hypothetical protein |
| PA14_19100 | 5.32 | 1.53 | *rhlA*, rhamnosyltransferase chain A |
| PA14_19110 | 4.32 | 1.92 | *rhlB*, rhamnosyltransferase chain B |
| PA14_19140 | - | -1.74 | *pheC,* cyclohexadienyl dehydratase |
| PA14_19150 | - | -1.80 | hypothetical protein |
| PA14_20890 | -0.99 | - | *rfaD*, epimerase |
| PA14_21030 | 1.87 | - | ATP-dependent Clp protease proteolytic subunit |
| PA14_21530 | 2.96 | 3.34 | ankyrin domain-containing protein |
| PA14_22320 | 7.44 | 6.44 | hypothetical protein |
| PA14_24860 | 2.68 | 2.13 | *snr1*, cytochrome c Snr1 |
| PA14_26750 | 1.60 | 1.39 | hypothetical protein |
| PA14_26980 | 1.54 | 1.58 | hypothetical protein |
| PA14_26990 | 4.25 | 4.12 | hypothetical protein |
| PA14_27330 | 1.89 | 2.58 | phospho-2-dehydro-3-deoxyheptonate aldolase |
| PA14_28050 | 1.29 | - | chemotaxis transducer |
| PA14_28250 | - | 1.72 | secreted acid phosphatase |
| PA14_28260 | 1.14 | 1.25 | hypothetical protein |
| PA14_28360 | 2.50 | 1.46 | hypothetical protein |
| PA14_30620 | 1.84 | 1.51 | AraC family transcriptional regulator |
| PA14_31290 | 3.54 | 2.76 | *pa1L*, PA-I galactophilic lectin |
| PA14_31350 | 1.80 | - | hypothetical protein |
| PA14_31360 | 2.72 | 1.88 | hypothetical protein |
| PA14_31370 | 2.49 | 1.61 | hypothetical protein |
| PA14_31840 | 1.44 | - | hypothetical protein |
| PA14_32830 | - | -1.04 | hypothetical protein |
| PA14_32950 | 2.53 | 2.33 | hypothetical protein |
| PA14_34870 | 4.63 | 3.83 | *chiC*, chitinase |
| PA14_35160 | 4.84 | 2.24 | hypothetical protein |
| PA14_35170 | 1.75 | - | redox-sensing activator of SoxS |
| PA14_36310 | 3.55 | - | *hcnC*, hydrogen cyanide synthase HcnC |
| PA14_36320 | 3.57 | - | *hcnB*, hydrogen cyanide synthase HcnB |
| PA14_36330 | 3.45 | - | *hcnA*, hydrogen cyanide synthase HcnA |
| PA14_37210 | -1.49 | - | hypothetical protein |
| PA14_37250 | -1.45 | - | major facilitator transporter |
| PA14_37260 | -1.80 | - | porin |
| PA14_37270 | -1.88 | - | LamB/YcsF family protein |
| PA14_37710 | 1.01 | - | *fusA2*, elongation factor G |
| PA14_37745 | 2.89 | 1.93 | carbamoyl transferase |
| PA14_37760 | 4.80 | 2.32 | MFS transporter |
| PA14_37770 | 3.25 | 2.59 | hydrolase |
| PA14_37780 | 3.09 | 1.88 | hypothetical protein |
| PA14_38260 | 2.73 | 2.26 | hypothetical protein |
| PA14_39460 | 1.62 | - | hypothetical protein |
| PA14_39780 | 1.33 | 1.40 | hypothetical protein |
| PA14_39960 | 5.63 | 3.18 | *phzB2*, phenazine biosynthesis protein |
| PA14_39970 | 4.28 | 2.48 | *phzA2*, phenazine biosynthesis protein |
| PA14_40290 | 2.36 | 2.05 | *lasA*, LasA protease |
| PA14_40310 | 2.65 | - | acyl carrier protein |
| PA14_43000 | 1.38 | - | *hsiG2*, HsiG2 |
| PA14_43030 | 1.31 | - | *hsiC2*, HsiC2 |
| PA14_43040 | 1.72 | - | *hsiB2*, HsiB2 |
| PA14_43050 | 1.25 | - | *hsiA2*, HsiA2 |
| PA14_48530 | 2.09 | - | AMP-binding protein |
| PA14_48560 | 2.97 | - | hypothetical protein |
| PA14_48600 | 1.98 | - | AMP-binding protein |
| PA14_49750 | 2.54 | - | MFS family transporter |
| PA14_49760 | 2.97 | - | *rhlC*, rhamnosyltransferase 2 |
| PA14_51350 | -2.12 | -1.91 | *phnB*, anthranilate synthase component II |
| PA14_51360 | -2.58 | -2.22 | *phnA*, anthranilate synthase component I |
| PA14_51380 | -2.55 | -2.27 | *pqsE*, quinolone signal response protein |
| PA14_51390 | -2.57 | -2.16 | *pqsD*, 3-oxoacyl-ACP synthase |
| PA14_51410 | -2.65 | -2.11 | *pqsC*, PqsC |
| PA14_51420 | -2.63 | -2.14 | *pqsB*, PqsB |
| PA14_51430 | -2.55 | -2.04 | *pqsA*, PqsA |
| PA14_51830 | 1.49 | - | DNA-binding stress protein |
| PA14_52130 | 1.35 | 1.07 | hypothetical protein |
| PA14_53250 | 3.33 | 2.90 | *cpbD*, chitin-binding protein CbpD |
| PA14_53290 | 3.05 | 3.09 | *trxB2*, thioredoxin reductase 2 |
| PA14_53300 | 5.73 | 5.33 | alkyl hydroperoxide reductase |
| PA14_53610 | - | -1.34 | hypothetical protein |
| PA14_56990 | 2.02 | - | hypothetical protein |
| PA14_58630 | 1.21 | 1.15 | ornithine decarboxylase |
| PA14_60560 | -0.99 | - | hypothetical protein |
| PA14_61040 | 2.14 | 2.28 | *katB*, catalase |
| PA14_61200 | - | 1.39 | hypothetical protein |
| PA14_62790 | -1.31 | -1.28 | tRNA-Met |
| PA14_63080 | -1.25 | - | lldP, L-lactate permease |
| PA14_64930 | 2.10 | - | hypothetical protein |
| PA14_64940 | 2.19 | - | hypothetical protein |
| PA14_66410 | 1.86 | - | hypothetical protein |
| PA14_66460 | 1.05 | 1.41 | hypothetical protein |
| PA14_66840 | 1.18 | - | *phaC2*, poly (3-hydroxyalkanoic acid) synthase 2 |
| PA14_68170 | 2.40 | 1.52 | *rmlB*, dTDP-D-glucose 4,6-dehydratase |
| PA14_68190 | 2.62 | 1.51 | *rmlD*, dTDP-4-dehydrorhamnose reductase |
| PA14_68200 | 2.60 | 1.68 | *rmlA*, glucose-1-phosphate thymidylyltransferase |
| PA14_68210 | 2.51 | 1.60 | *rmlC*, dTDP-4-dehydrorhamnose 3,5-epimerase |
| PA14_68230 | 1.73 | 1.10 | two-component sensor |
| PA14_68930 | 3.02 | - | permease |
| PA14_68940 | 3.94 | 2.13 | hypothetical protein |
| PA14_69190 | -1.09 | - | *rho*, transcription termination factor Rho |
| PA14_69200 | - | 1.01 | *trxA*, thioredoxin |
| PA14_69250 | -1.06 | - | hypothetical protein |
| PA14_70860 | -1.09 | - | hypothetical protein |

a: HCD refers to high cell density: OD_600_ ~2.0

b: annotation taken from pseudomonas.com [85]

c: Three categories of RhlR-regulated genes are:  Class I, gray, Class II, yellow, and Class III, cyan

d: at least +/- 1 log_2_-fold regulated genes with P-values < 0.001 are shown

e: dashes (-) indicate either no regulation by RhlR or no regulation by RhlI
