# Supplementary material for: The RhlR quorum-sensing receptor controls Pseudomonas aeruginosa pathogenesis and biofilm development independently of its canonical homoserine lactone autoinducer
Source: PLoS Pathog. 2017 Jul 17;13(7):e1006504. doi: 10.1371/journal.ppat.1006504 (PMC5531660; doi:10.1371/journal.ppat.1006504)
Supplement: S2 Table — (DOCX) [file ppat.1006504.s013.docx]

**Table S2. Genes controlled in *P. aeruginosa* PA14 by RhlR and RhlI in colony biofilms determined by RNA-seq.**

| **PA14 ID^a^** | **log_2_-fold change^b,c^** | | **Gene name and description** |
| --- | --- | --- | --- |
|  | **Δ*rhlR*** | **Δ*rhlI*** |  |
| PA14_00450 | -2.33 | - | *trpB*, tryptophan synthase subunit beta |
| PA14_00470 | -1.34 | - | hypothetical protein |
| PA14_00630 | 1.76 | - | hypothetical protein |
| PA14_00640 | 4.55 | - | *phzH*, potential phenazine-modifying enzyme |
| PA14_00650 | 1.59 | - | hypothetical protein |
| PA14_01320 | - | 1.29 | *coIII*, cytochrome c oxidase subunit III |
| PA14_01490 | 5.06 | - | hemolysin |
| PA14_02520 | 1.29 | - | hypothetical protein |
| PA14_03490 | 2.87 | - | hypothetical protein |
| PA14_05660 | 2.05 | - | transcriptional regulator |
| PA14_06960 | 2.03 | - | hypothetical protein |
| PA14_07030 | 1.58 | - | cytochrome c' |
| PA14_07110 | 1.99 | - | ArsR family transcriptional regulator |
| PA14_09400 | 2.67 | -2.70 | *phzS*, hypothetical protein |
| PA14_09490 | 1.47 | -2.78 | *phzM*, phenazine-specific methyltransferase |
| PA14_09520 | 3.38 | - | *mexI*, RND efflux transporter |
| PA14_09530 | 5.17 | - | *mexH*, RND efflux membrane fusion protein |
| PA14_09540 | 3.97 | - | *mexG*, hypothetical protein |
| PA14_10350 | 2.38 | - | secretion protein |
| PA14_10360 | 3.78 | - | hypothetical protein |
| PA14_11140 | 3.89 | - | nonribosomal peptide synthetase |
| PA14_13170 | 1.86 | - | metal transporting P-type ATPase |
| PA14_13210 | 3.13 | - | hypothetical protein |
| PA14_13280 | 2.64 | - | *moeA1*, molybdenum cofactor biosynthetic protein A1 |
| PA14_13750 | 2.30 | - | *narK1*, nitrite extrusion protein 1 |
| PA14_14660 | -1.64 | - | hypothetical protein |
| PA14_15050 | 1.52 | - | hypothetical protein |
| PA14_16250 | 2.51 | - | *lasB*, elastase LasB |
| PA14_16310 | 3.21 | - | MFS permease |
| PA14_18800 | 4.66 | - | hypothetical protein |
| PA14_19100 | 2.81 | - | *rhlA*, rhamnosyltransferase chain A |
| PA14_19110 | 4.10 | - | *rhlB*, rhamnosyltransferase chain B |
| PA14_19140 | 1.30 | - | *pheC*, cyclohexadienyl dehydratase |
| PA14_20560 | 1.46 | - | *amiE*, acylamide amidohydrolase |
| PA14_20610 | 2.86 | - | *lecB*, fucose-binding lectin PA-IIL |
| PA14_20900 | 4.16 | - | MFS transporter |
| PA14_20920 | 1.82 | - | hypothetical protein |
| PA14_20950 | 4.72 | - | *fabH2*, 3-oxoacyl-ACP synthase |
| PA14_20970 | 3.55 | - | *cyp23*, cytochrome P450 |
| PA14_20980 | 2.49 | - | short chain dehydrogenase |
| PA14_21020 | 3.06 | -1.21 | non-ribosomal peptide synthetase |
| PA14_21470 | 2.70 | - | hypothetical protein |
| PA14_21630 | -1.04 | - | hypothetical protein |
| PA14_24560 | 2.12 | 2.13 | *pelG*, hypothetical protein |
| PA14_25900 | -1.40 | - | trans-2-enoyl-CoA reductase |
| PA14_27630 | -2.16 | - | protein associated with synthesis and  assembly of refractile inclusion bodies |
| PA14_28050 | 1.59 | - | chemotaxis transducer |
| PA14_28250 | 2.89 | 1.82 | secreted acid phosphatase |
| PA14_28360 | 2.65 | - | hypothetical protein |
| PA14_29660 | 1.28 | - | hypothetical protein |
| PA14_29760 | - | -2.26 | chemotaxis transducer |
| PA14_30050 | -1.52 | - | isocitrate lyase |
| PA14_30190 | -1.39 | - | Icd, isocitrate dehydrogenase |
| PA14_30240 | 1.25 | - | *infA*, translation initiation factor IF-1 |
| PA14_30570 | 1.80 | - | periplasmic spermidine/putrescine-binding protein |
| PA14_30620 | 1.82 | - | AraC family transcriptional regulator |
| PA14_30630 | 1.34 | - | *pqsH*, FAD-dependent monooxygenase |
| PA14_31290 | 6.16 | 2.34 | *pa1L*, PA-I galactophilic lectin |
| PA14_31350 | 2.70 | - | hypothetical protein |
| PA14_31360 | 3.37 | - | hypothetical protein |
| PA14_31370 | 2.33 | - | hypothetical protein |
| PA14_33160 | -2.37 | - | hypothetical protein |
| PA14_33250 | -1.91 | - | hypothetical protein |
| PA14_33280 | -1.29 | - | *pvdL*, peptide synthase |
| PA14_33510 | -1.32 | - | hypothetical protein |
| PA14_33610 | -1.28 | - | peptide synthase |
| PA14_33690 | -1.40 | - | *pvdE*, pyoverdine biosynthesis protein PvdE |
| PA14_33730 | -1.32 | - | dipeptidase |
| PA14_34870 | 6.04 | 1.88 | *chiC*, chitinase |
| PA14_35160 | 2.52 | - | hypothetical protein |
| PA14_36310 | 3.44 | - | *hcnC*, hydrogen cyanide synthase HcnC |
| PA14_36320 | 2.68 | - | *hcnB*, hydrogen cyanide synthase HcnB |
| PA14_36330 | 3.17 | - | *hcnA*, hydrogen cyanide synthase HcnA |
| PA14_36450 | -2.10 | - | hypothetical protein |
| PA14_36910 | -1.83 | - | *ligD*, ATP-dependent DNA ligase |
| PA14_37360 | 2.76 | - | short chain dehydrogenase |
| PA14_37380 | 2.95 | - | flavin-binding monooxygenase |
| PA14_37745 | 6.02 | - | carbamoyl transferase |
| PA14_37760 | 6.87 | - | MFS transporter |
| PA14_37770 | 2.99 | - | hydrolase |
| PA14_37780 | 3.51 | - | hypothetical protein |
| PA14_38220 | -1.68 | - | hypothetical protein |
| PA14_39460 | 2.45 | - | hypothetical protein |
| PA14_39590 | 2.13 | - | *metE*, homocysteine S-methyltransferase |
| PA14_39780 | 3.05 | - | hypothetical protein |
| PA14_39970 | 4.44 | - | *phzA2*, phenazine biosynthesis protein |
| PA14_40260 | 1.04 | - | hypothetical protein |
| PA14_40310 | 6.03 | - | acyl carrier protein |
| PA14_40560 | -1.39 | - | hypothetical protein |
| PA14_41450 | 1.59 | - | hypothetical protein |
| PA14_42080 | - | -2.003 | 3-hydroxyacyl-CoA dehydrogenase |
| PA14_42090 | - | -1.82 | acetyl-CoA acetyltransferase |
| PA14_42900 | 2.24 | - | *icmF2*, IcmF2 |
| PA14_42990 | 2.63 | - | *hsiH2*, HsiH2 |
| PA14_43000 | 3.04 | - | *hsiG2*, HsiG2 |
| PA14_43030 | 1.49 | - | *hsiC2*, HsiC2 |
| PA14_43040 | 1.73 | - | *hsiB2*, HsiB2 |
| PA14_43050 | 2.16 | - | *hsiA2*, HsiA2 |
| PA14_44420 | 1.15 | - | ferredoxin |
| PA14_45370 | 1.52 | - | *ccmB*, heme exporter protein CcmB |
| PA14_48040 | 1.79 | - | *aprI* ,alkaline proteinase inhibitor AprI |
| PA14_49050 | -1.98 | - | hypothetical protein |
| PA14_49300 | - | 1.75 | lipoxygenase |
| PA14_49310 | - | 1.86 | hypothetical protein |
| PA14_49750 | 6.97 | - | MFS family transporter |
| PA14_51350 | -2.62 | -2.28 | *phnB*, anthranilate synthase component II |
| PA14_51360 | -3.07 | -2.16 | *phnA*, anthranilate synthase component I |
| PA14_51380 | -3.53 | -2.64 | *pqsE*, quinolone signal response protein |
| PA14_51390 | -3.43 | -2.62 | *pqsD*, 3-oxoacyl-ACP synthase |
| PA14_51410 | -3.18 | -2.64 | *pqsC*, PqsC |
| PA14_51420 | -3.23 | -2.89 | *pqsB*, PqsB |
| PA14_51430 | -3.13 | -2.37 | *pqsA*, PqsA |
| PA14_52130 | 2.10 | - | hypothetical protein |
| PA14_53250 | 4.34 | 2.20 | *cpbD*, chitin-binding protein CbpD |
| PA14_54420 | -2.05 | - | *mucA*, anti-sigma factor MucA |
| PA14_54430 | -1.77 | - | *algU*, RNA polymerase sigma factor AlgU |
| PA14_55940 | 2.02 | - | hypothetical protein |
| PA14_56590 | 2.23 | - | hypothetical protein |
| PA14_56990 | 3.70 | - | hypothetical protein |
| PA14_58500 | -2.65 | - | hypothetical protein |
| PA14_58630 | 1.67 | - | ornithine decarboxylase |
| PA14_60400 | 1.22 | - | *rpsT*, 30S ribosomal protein S20 |
| PA14_61760 | 1.24 | - | tRNA-Gln |
| PA14_61770 | 1.49 | - | *prs*, ribose-phosphate pyrophosphokinase |
| PA14_64940 | 3.47 | 1.98 | hypothetical protein |
| PA14_66460 | 2.81 | - | hypothetical protein |
| PA14_66710 | 1.16 | - | *rpmE*, 50S ribosomal protein L31 |
| PA14_66840 | 1.96 | - | *phaC2*, poly (3-hydroxyalkanoic acid) synthase 2 |
| PA14_66850 | 1.58 | - | TetR family transcriptional regulator |
| PA14_67180 | 1.99 | - | hypothetical protein |
| PA14_67190 | 3.26 | - | hypothetical protein |
| PA14_68170 | 3.83 | 1.57 | *rmlB*, dTDP-D-glucose 4,6-dehydratase |
| PA14_68190 | 2.60 | - | *rmlD*, dTDP-4-dehydrorhamnose reductase |
| PA14_68200 | 2.22 | - | *rmlA*, glucose-1-phosphate thymidylyltransferase |
| PA14_68210 | 2.59 | 1.21 | *rmlC*, dTDP-4-dehydrorhamnose 3,5-epimerase |
| PA14_68230 | 1.67 | - | two-component sensor |
| PA14_68930 | 5.05 | - | permease |
| PA14_68940 | 5.33 | - | hypothetical protein |
| PA14_69795 | 1.77 | - | *amtB*, ammonium transporter |
| PA14_69990 | 2.58 | - | *dadX*, alanine racemase |
| PA14_70690 | 2.32 | - | *glcD*, glycolate oxidase subunit GlcD |
| PA14_72360 | -1.79 | - | hypothetical protein |
| PA14_72900 | -1.39 | - | lipoprotein |

a: annotation taken from pseudomonas.com [85]

b: at least +/- 1 log_2_-fold regulated genes with P-values < 0.001 are shown

c: dashes (-) indicate either no regulation by RhlR or no regulation by RhlI
