# Supplementary material for: The RhlR quorum-sensing receptor controls Pseudomonas aeruginosa pathogenesis and biofilm development independently of its canonical homoserine lactone autoinducer
Source: PLoS Pathog. 2017 Jul 17;13(7):e1006504. doi: 10.1371/journal.ppat.1006504 (PMC5531660; doi:10.1371/journal.ppat.1006504)
Supplement: S3 Table — (DOCX) [file ppat.1006504.s014.docx]

| **Strain or plasmid** | **Description** | **Reference** |
| --- | --- | --- |
| UCBPP-PA14 | Wild type, generous gift from Dr. George O’Toole | [70] |
| SM32 | Δ*rhlR* | [40,41] and this study |
| SM51 | Δ*lasI* | [40] and this study |
| SM52 | Δ*rhlI* | [40] and this study |
| SM67 | Δ*lasR* | [40,41] and this study |
| SM149 | Δ*rhlA^C11STOP^* | This study |
| SM150 | Δ*rhlR* p*rhlR* | This study |
| SM159 | Δ*rhlR^W11STOP^* | This study |
| SM173 | Δ*rhlR* pUCP18 | This study |
| SM367 | Wild type *P1-lux* | This study |
| SM368 | Δ*rhlR P1-lux* | This study |
| SM369 | Δ*rhlI P1-lux* | This study |
| SM370 | Δ*lasR P1-lux* | This study |
| SM371 | Δ*lasI P1-lux* | This study |
| SM381 | *PrhlA-mNeonGreen* | [41] and this study |
| SM383 | Δ*rhlR PrhlA-mNeonGreen* | [41] and this study |
| SM384 | Δ*rhlI PrhlA-mNeonGreen* | This study |
| SM391 | Δ*lasR* Δ*lasI* Δ*rhlR* Δ*rhlI* (Δ4) *PrhlA-mNeonGreen* | This study |
| SM392 | Δ*lasI PrhlA-mNeonGreen* | This study |
| SM393 | Δ*lasR PrhlA-mNeonGreen* | [41] and this study |
| SM401 | Δ4 *P_BAD_-rhlR PrhlA-mNeonGreen* | This study |
| SM419 | Δ*rhlI* Δ*pelA* | This study |
| SM421 | Δ*rhlR* Δ*pelA* | This study |
| SM466 | Δ*rhlI^F50STOP^* | This study |
| SM645 | Δ*phz* | This study |
| SM652 | Δ*rhlA^C11STOP^* Δ*rhlR* | This study |
| SM654 | Δ*rhlA^C11STOP^* Δ*rhlI* | This study |
| SM656 | Δ*rhlR* Δ*phz* | This study |
| SM659 | Δ*rhlI* Δ*phz* | This study |
| *E.coli* DH5α | F^–^ *endA1* *glnV44* *thi-1* *recA1* *relA1* *gyrA96* *deoR* *nupG* *purB20* φ80d*lacZ*ΔM15 Δ(*lacZYA-argF*)U169, hsdR17(*r_K_*^–^*m_K_*^+^), λ^–^ | Laboratory stock |
| *E.coli* SM10λ*pir* | *thi thr leu tonA lacY supE recA*::RP4-2-Tc::Mu | Laboratory stock |
| *E.coli* OP50 | Uracil-requiring mutant of *E.coli* | [86] |
| pEXG2 | Allelic exchange vector with pBR origin, gentamicin resistance, *sacB,* generous gift from Dr. Joseph Mougous | [78] |
| pUCP18 | E. coli-Pseudomonas Amp^r^ shuttle vector | [79] |
| pTJ1 | pUC18T-mini-Tn*7*T-Tp-*araC*-PBAD-MCS | [81] |
| pUC18T-mini-Tn*7*T-*lux-*Tp | *dhfrIIb oriT P1*-*luxCDABE*; Amp^r^ Tp^r^ | [83] |

**S3 Table: Bacterial strains and plasmids**
